# Novel antibiotics effective against gram-positive and -negative multi-resistant bacteria with limited resistance

**DOI:** 10.1371/journal.pbio.3000337

**Published:** 2019-07-09

**Authors:** Irène Nicolas, Valérie Bordeau, Arnaud Bondon, Michèle Baudy-Floc’h, Brice Felden

**Affiliations:** 1 University of Rennes, Inserm, BRM [Bacterial Regulatory RNAs and Medicine] UMR_S 1230, Rue du Professeur Léon Bernard, France; 2 University of Rennes, CNRS, ISCR–UMR 6226, France; Biological Research Center, HUNGARY

## Abstract

Antibiotics are a medical wonder, but an increasing frequency of resistance among most human pathogens is rendering them ineffective. If this trend continues, the consequences for public health and for the general community could be catastrophic. The current clinical pipeline, however, is very limited and is dominated by derivatives of established classes, the “me too” compounds. Here, we have exploited our recent identification of a bacterial toxin to transform it into antibiotics active on multidrug-resistant (MDR) gram-positive and -negative bacterial pathogens. We generated a new family of peptidomimetics—cyclic heptapseudopeptides—inspired from a natural bacterial peptide. Out of the 4 peptides studied, 2 are effective against methicillin-resistant *Staphylococcus aureus* (MRSA) in mild and severe sepsis mouse models without exhibiting toxicity on human erythrocytes and kidney cells, zebrafish embryos, and mice. These new compounds are safe at their active doses and above, without nephrotoxicity. Efficacy was also demonstrated against *Pseudomonas aeruginosa* and MRSA in a mouse skin infection model. Importantly, these compounds did not result in resistance after serial passages for 2 weeks and 4 or 6 days’ exposure in mice. Activity of heptapseudopeptides was explained by the ability of unnatural amino acids to strengthen dynamic association with bacterial lipid bilayers and to induce membrane permeability, leading to bacterial death. Based on structure determination, we showed that cationic domains surrounded by an extended hydrophobic core could improve bactericidal activity. Because 2 peptide analogs, Pep 16 and Pep19, are effective against both MRSA and *P*. *aeruginosa* in severe sepsis and skin infection models, respectively, we believe that these peptidomimetics are promising lead candidates for drug development. We have identified potential therapeutic agents that can provide alternative treatments against antimicrobial resistance. Because the compounds are potential leads for therapeutic development, the next step is to start phase I clinical trials.

## Abstract 2

A naturally occurring bacterial toxin inspired the design of novel cyclic heptapseudopeptide antibiotics with limited resistance; these new peptidomimetics are active against gram-negative and -positive multi-resistant ESKAPE pathogens.

## Introduction

With time, widespread bacterial antimicrobial resistance diminishes the clinical efficacy of antibiotics, threating the health of humans and animals [1,2]. There is serious concern about the rise of antibiotic-resistant “superbugs” now resistant to many antibiotics [3]. These include the ESKAPE pathogens (*Enterococcus faecium*, *Staphylococcus aureus*, *Klebsiella pneumoniae*, *Acinetobacter baumannii*, *Pseudomonas aeruginosa*, and *Enterobacter* spp.) [4]. The dramatic increase in antibiotic resistance makes infectious diseases a challenge for medicine worldwide. The total deaths from methicillin-resistant *S*. *aureus* (MRSA) are now comparable to those caused by HIV, and it is estimated that by the year 2050, at least 10 million people will die annually due to antimicrobial resistance [5]. The endless evolution and spread of antibiotic resistance and the emergence of new pathogens are driving the quest for novel drugs, now an urgent necessity [6].

Antimicrobial peptides (AMPs) are essential components of innate immunity in most organisms that fight against microbial challenges. Although some AMPs are templates for developing new antibiotics [7], progress is restricted by their toxicity and reduced half-lives in human body fluids. When secreted, small bacterial toxins can act as AMPs. Their toxicity to human cells can be reduced while retaining their antibiotic activity [8].

**Fig 1:**
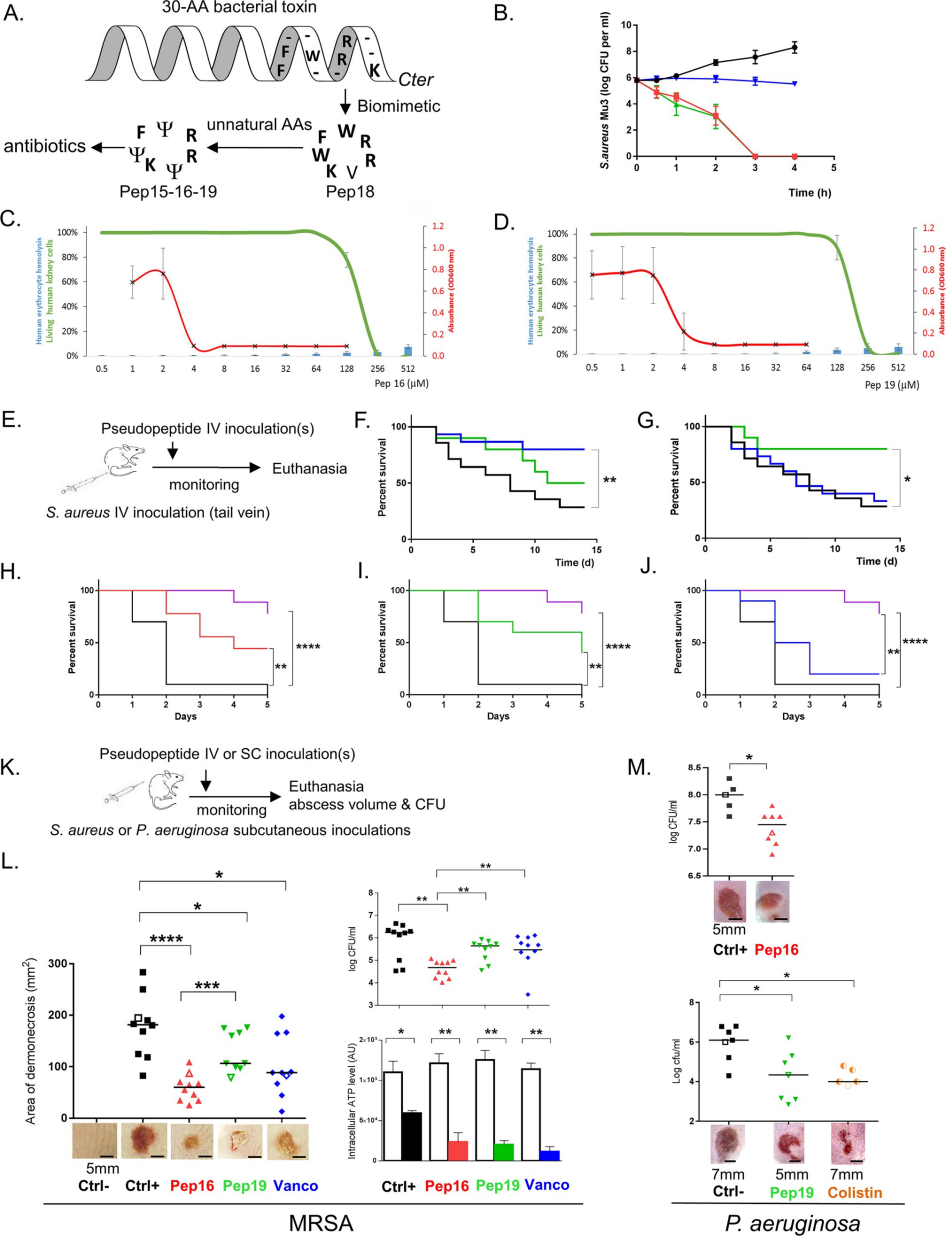
Two pseudopeptides are active against gram-positive and -negative MDR bacteria in murine sepsis and skin infection models and are nontoxic. (A) Biomimetic design of antibiotics from a bacterial toxin [9]. Selected residues from the *S*. *aureus* toxin were cyclized, and unnatural amino acids (Ψ) were incorporated to synthesize those new antibiotics. Data associated with this panel can be found in S1 Fig. (B) Kill curves of MRSA using Pep16 (red), Pep19 (green), and vancomycin (blue) at concentrations 30-fold above the MIC are compared to untreated bacteria (black). Means and standard errors of the means are representative of 3 independent biological replicates. Data associated with this panel can be found in S3 Fig. (C–D) Pep16 and Pep19 antibiotic activity against MRSA strain Mu3 (red) compared to their low toxicity on human erythrocytes (blue bars) or to human kidney cell viability (green). Pep16 and Pep19 concentration range is from 0.5 to 512 μM. Data associated with this panel can be found in S4 Fig and S2–S6 Tables. (E) Overview of the mouse sepsis experiment. (F–G) Kaplan-Meier survival probability plots of mice infected with either MRSA without treatment (black), or with 1.5 mg.kg^−1^ Pep19 (green) or vancomycin (blue). In a sepsis protection model, groups of 5 Swiss mice were inoculated with approximately 5 × 10^8^ MRSA and treated either 3 h (F) or 15 h (G) post infection. Survival was monitored for 14 days after infection (panel d; x-axis), and the results are from 10 mice. The experiment was performed twice and the data combined. Data associated with these two panels can be found in S3 Fig. (H–J) Kaplan-Meier survival probability plots of severe sepsis assays (nearly all untreated infected mice killed within 2 days post infection) with mice infected with approximately 2.10^9^ CFUs MRSA either without treatment (black) or with 4 repeated doses (0.5 mg.kg^−1^) of Pep16 (red), Pep19 (green), vancomycin (blue), or brilacidin (purple). The results are from 5 mice per assay, and the experiment was performed twice. Mice monitoring was performed for 5 days. (K) Overview of the mouse skin infection experiments with either *S*. *aureus* or *P*. *aeruginosa*. (L) Treatment of cutaneous abscesses from MRSA in mice using Pep16, Pep19, or vancomycin. Mice were infected with approximately 2 × 10^9^ MRSA and treated by IV 24 h post infection with a saline solution (black) or with 1.5 mg.kg^−1^ of Pep16 (red) or Pep19 (green) or vancomycin (blue). Lesion sizes and the CFU counts per abscess, plotted as individual points, were determined 6 days post infection (10 mice per condition). The abscess photos below the graph correspond to each experimental value shown in the graph as empty symbols. Intracellular ATP levels from *S*. *aureus* in the “normal-growing” (empty histograms) versus SCVs (filled colored histograms) collected from the mice skin abscesses (lower panels). (M) Treatment of cutaneous abscesses induced by *P*. *aeruginosa* in mice using Pep16, Pep19, or colistin. Mice were infected with approximately 108 *P*. *aeruginosa* and treated with repeated doses post infection with a saline solution (black) or with Pep16 (red, 30 mg.kg^−1^), Pep19 (green, 30 mg.kg^−1^), or colistin (orange, 9 mg.kg^−1^, lower panel). The CFU counts per abscess, plotted as individual points, were determined 3 days post infection (5 to 8 mice per condition). The abscess photos below the graph correspond to each experimental value shown as empty symbols. Mann-Whitney was used to calculate the differences between the groups; *0.05 < *P* < 0.01; **0.01 < *P* < 0.001; ***0.001 < *P* < 0.0001; ****P < 0.00001. Data associated with this figure can be found in S1 Data. CFU, colony-forming unit; Ctrl, controls; MDR, multidrug resistant; MIC, minimal inhibitory concentration; MRSA, methicillin-resistant *S*. *aureus*; OD600, optical density at 600 nm; SCV, small colony variant.

In the present study, we designed and synthesized 4 cyclic heptapseudopeptide biomimetics (Fig 1A and S1 Fig), inspired by and imitating a section of an *S*. *aureus* toxin, PepA1 [9]. PepA1 is a toxic linear peptide expressed from a type I toxin-antitoxin system the synthesis of which is repressed by an antisense RNA during bacterial growth. Herein, we designed novel pseudopeptides with activities against antibiotic-resistant gram-positive and -negative pathogens in infected mice, with limited potential of resistance emergence.

## Results

### Antibiotic activity, emergence of resistance and toxicity

We first tested their bactericidal effects against a broad range of gram-positive and -negative pathogens, including multidrug-resistant (MDR) human isolates from bloodstream, urinary, and respiratory tract infections (Table 1 for ESKAPE clinical isolates, and S1 Table for several *S*. *aureus* MDR clinical isolates). All 4 pseudopeptides showed bactericidal effects against all the tested clinical isolates, but 3 of them (Pep15, Pep16, and Pep19) contained aza-β3-amino acid analogs that enhance antimicrobial activity (S1 Fig) [10,11]. Indeed, higher bactericidal activity was observed for the 3 macromolecules containing amino acid analogs compared to the molecule containing only natural amino acids (Pep18). Antibiograms of several clinical isolates included in the minimum inhibitory concentration (MIC) assay are provided (S2 Fig), showing that they are MDR bacteria. Furthermore, we compared the activity of these pseudopeptides to that of antibiotics in clinical use against MRSA Mu3. The MRSA time-kill curve natural amino acids comparisons show that Pep16, Pep18, and Pep19 have higher bactericidal activities than vancomycin (Fig 1B and S3A Fig)—a slow bactericidal agent but a standard molecule used for treatment of systemic MRSA infections [12].

**Table 1:** Spectrum of activity of the peptidomimetics and standard antibiotics against the ESKAPE pathogens.

		Pep15		Pep16		Pep18		Pep19		Vancomycin		Polymyxin B		Colistin		Daptomycin		Methicillin	Nisin
	Strains	MIC μM (mg.L^−1^)	MBC/MIC	MIC μM (mg.L^−1^)	MBC/MIC	MIC μM (mg.L^−1^)	MBC/MIC	MIC μM (mg.L^−1^)	MBC/MIC	MIC μM (mg.L^−1^)	MBC/MIC	MIC μM (mg.L^−1^)	MBC/MIC	MIC μM (mg.L^−1^)	MBC/MIC	MIC μM (mg.L^−1^)	MBC/MIC	MIC μM (mg.L^−1^)	MIC μM (mg.L^−1^)
**Gram+ ESKAPE**	***S*. *aureus***																		
	Newman	4 (4.6)	2	4 (4.5)	2	16 (16.4)	2	4 (4.5)	2	0.5 (0.7)	2	-	*-*	-	-	0.25 (0.5)	1	-	8 (32)
	Mu3*	2 (2.3)	2	4 (4.5)	2	8 (8.2)	2	4 (4.5)	2	1 (1.4)	1	-	*-*			0.5 (1)	1	>512 mM >190 g.L^−1^	8 (32)
	N315*	2 (2.3)	2	4 (4.5)	2	8 (8.2)	2	4 (4.5)	2	2 (2.8)	-	-	*-*	-	-	-	-	32 (16)	8 (32)
	***E*. *faecium***																		
	Aus0004	8 (9.2)	4	8 (9)	2	64 (65.6)	2	8 (9)	2	**-**	-	-	*-*	-	-	0.5 (1)	1	-	-
**Gram− ESKAPE**	***Escherichia coli***																		
	K12	2 (2.3)	1	4 (4.5)	1	16 (16.4)	1	4 (4.5)	1	-	*-*	0.5 (0.65)	1	0.5 (0.65)	1	-	*-*	*-*	4 (16)
	ML35P	4 (4.6)	1	8 (9)	1	16 (16.4)	1	4 (4.5)	1	-	*-*	0.5 (0.65)	1	0.5 (0.65)	1	-	*-*	*-*	-
	13090**	4 (4.6)	1	4 (4.5)	1	16 (16.4)	1	4 (4.5)	1	-	*-*	0.5 (0.65)	1	1 (1.3)	1	-	*-*	*-*	-
	PV****	8 (9.2)	1	8 (9)	1	32 (32.8)	1	8 (9)	1	-	*-*	0.5 (0.65)	1	-	*-*	-	*-*	*-*	-
	758***	8 (9.2)	1	8 (9)	1	32 (32.8)	1	8 (9)	1	-	*-*	0.5 (0.65)	1	-	*-*	-	*-*	*-*	-
	283***	8 (9.2)	1	8 (9)	1	32 (32.8)	1	8 (9)	1	-	*-*	0.5 (0.65)	1	-	*-*	-	*-*	*-*	-
	508***	8 (9.2)	1	8 (9)	1	64 (65.6)	1	8 (9)	1	-	*-*	0.5 (0.65)	1	-	*-*	-	*-*	*-*	-
	444***	8 (9.2)	1	8 (9)	1	32 (32.8)	1	8 (9)	1	-	*-*	0.5 (0.65)	1	-	*-*	-	*-*	*-*	-
	066***	8 (9.2)	1	8 (9)	1	32 (32.8)	1	8 (9)	1	-	*-*	1 (1.3)	1	-	*-*	-	*-*	*-*	-
	750***	8 (9.2)	1	8 (9)	1	16 (16.4)	1	8 (9)	1	-	*-*	1 (1.3)	1	-	*-*	-	*-*	*-*	-
	146***	8 (9.2)	1	8 (9)	1	32 (32.8)	1	8 (9)	1	-	*-*	1 (1.3)	1	-	*-*	-	*-*	*-*	-
	293***	8 (9.2)	1	8 (9)	1	32 (32.8)	1	16 (18)	1	-	*-*	0.5 (0.65)	1	-	*-*	-	*-*	*-*	-
	MCR−1 **	8 (9.2)	1	8 (9)	1	32 (32.8)	1	16 (18)	1	-	*-*	2 (2.6)	1	-	*-*	-	*-*	*-*	-
	***P*. *aeruginosa***																		
	PA14	8 (9.2)	2	4 (4.5)	4	8 (8.2)	4	8 (9)	2	-	*-*	0.5 (0.65)	2	0.5 (0.65)	2	-	*-*	*-*	-
	15637**	8 (9.2)	4	8 (9)	4	8 (8.2)	4	8 (9)	4	-	*-*	1 (1.3)	2	-	*-*	-	*-*	*-*	-
	15638**	16 (18.4)	2	32 (36)	2	64 (65.6)	2	16 (18)	2	-	*-*	1 (1.3)	2	-	*-*	-	*-*	*-*	-
	15641**	16 (18.4)	2	16 (18)	2	32 (32.8)	1	16 (18)	2	-	*-*	1 (1.3)	2	-	*-*	-	*-*	*-*	-
	15643**	8 (9.2)	4	8 (9)	4	16 (16.4)	4	8 (9)	4	-	*-*	0.5 (0.65)	2	0.5 (0.65)	2	-	*-*	*-*	-
	15644**	16 (18.4)	2	16 (18)	2	64 (65.6)	2	16 (18)	2	-	*-*	1 (1.3)	2	-	*-*	-	*-*	*-*	-
	***Enterobacter cloacae***																		
	ATCC13047	64 (73.6)	2	128 (144)	1	128 (131.2)	1	16 (18)	4	-	*-*	8 (10.4)	2	-	*-*	-	*-*	*-*	-
	***K*. *pneumoniae***																		
	13829**	> 128	-	>128	-	>128	-	>128	-	-	*-*	8 (10.4)	2	-	*-*	-	*-*	*-*	-
	13825**	16 (18.4)	4	16 (18)	4	16 (16.4)	4	8 (9)	2	-	*-*	2 (2.6)	2	-	*-*	-	*-*	*-*	-
	***A*. *baumanii***																		
	11896**	16 (18.4)	1	8 (9)	1	>128	*-*	8 (9)	1	-	*-*	1 (1.3)	2	-	*-*	-	*-*	*-*	-
	7985**	16 (18.4)	1	8 (9)	1	>128	*-*	8 (9)	1	-	*-*	1 (1.3)	1	-	*-*	-	*-*	*-*	-

* MRSA strains

**Clinical strains from Caen University Hospital hemocultures.

***Clinical strains from Rennes Hospital urinary catheters.

****Clinical strains from Rennes University Hospital vascular prosthetics.

**Fig 2:**
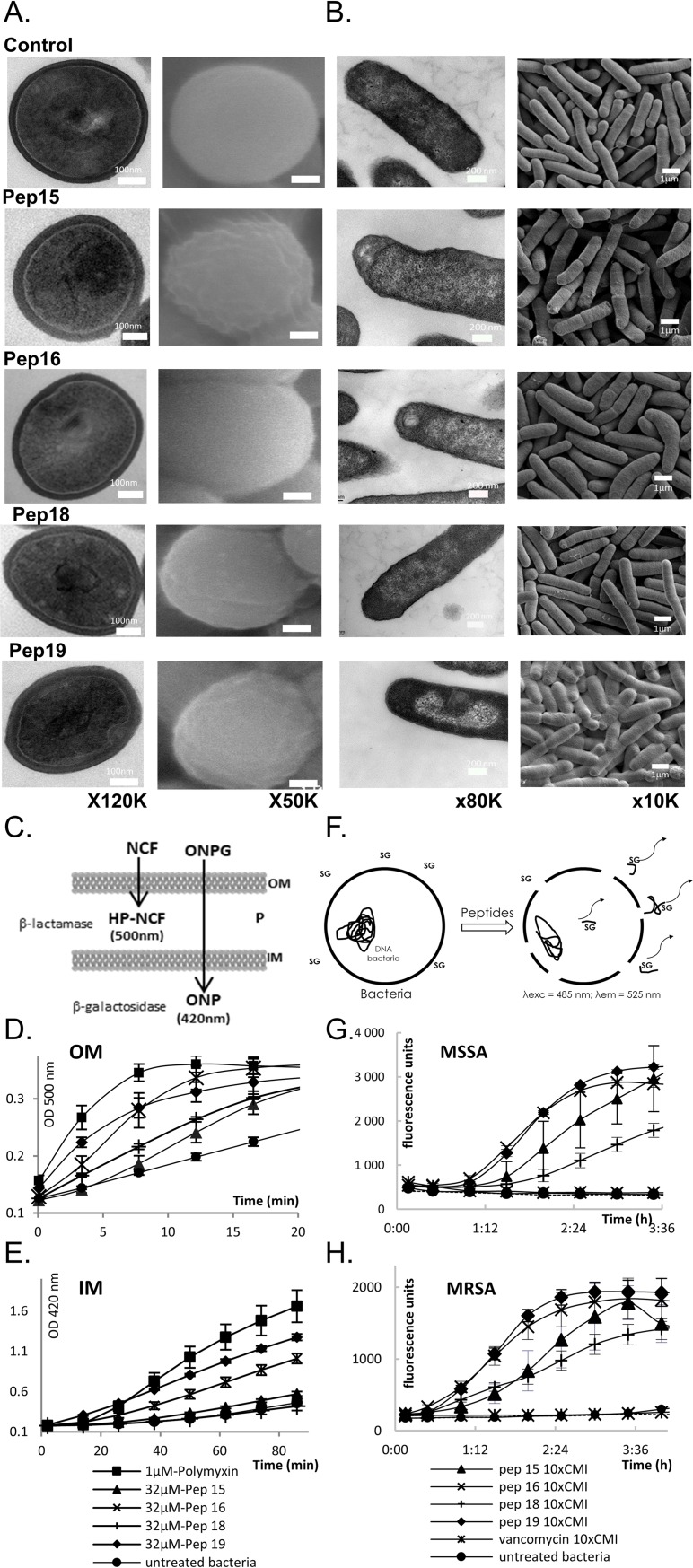
Pseudopeptide actions on gram-negative and -positive bacteria. Cell wall and membrane alterations of *S*. *aureus* Newman (A) and *E*. *coli* K12 (B), suggested by typical and representative TEM (left) and SEM (right) micrographs after treatment for 2 h at 37°C with Pep15, Pep16, Pep18, or Pep19 at their MICs. Untreated bacteria are unaltered, and cell walls and membranes remain intact. Pep15 and Pep19 erode cell walls (black arrows), and Pep19 triggers intracellular perturbations (white arrow). Data associated with panel (A) can be found in S6 Fig. Overview panels of the leakage assays for *E*. *coli* (C) and *S*. *aureus* (F). (C–H) Peptidomimetics permeate gram-negative (*E*. *coli*) OMs and IMs (panels C–E) and gram-positive MSSA and MRSA membranes (panels F–H). (C) Overview of the leakage experiments on gram-negative bacteria. (D) NCF degradation products triggered by periplasmic β-lactamases. (E) ONPG degradation products produced by cytoplasmic β-galactosidases. The mean values of 3 independent experiments are presented. Shown are polymyxin B as positive control, untreated bacteria, and Pep15, Pep16, Pep18, or Pep19. (F) Overview of the leakage experiments on gram-positive MSSA and MRSA. SG cannot enter intact cells but bind DNA and fluoresce once the cell integrity is damaged. MSSA (panel G) and MRSA (panel H) leakage experiments evidencing pseudopeptide-induced membrane alterations. On both gram-negative and -positive bacteria, the highest activity is with Pep16 and Pep19. Data associated with this figure can be found in S1 Data. IM, inner membrane; MIC, minimal inhibitory concentration; MRSA, methicillin-resistant *S*. *aureus*; MSSA, methicillin-susceptible *S*. *aureus*; NCF, nitrocefin; OM, outer membrane; ONPG, O-nitrophenyl-β-D-galactopyranoside; SEM, scanning electron microscopy; SG, sytox green; TEM, transmission electron microscopy.

The MBC/MIC ratios are provided. Concentrations are provided in μM and corresponding mg. L^−1^ in parentheses. The data are representative of 3 independent experiments. Data associated with this Table can be found in S1 Table and Fig 2.

**Abbreviations:** “-,” undetermined; ESKAPE, *Enterococcus faecium*, *S*. *aureus*, *Klebsiella pneumoniae*, *Acinetobacter baumannii*, *P*. *aeruginosa*, and *Enterobacter* spp.; MBC, minimal bactericidal concentration; MIC, minimal inhibitory concentration; MRSA, methicillin-resistant *S*. *aureus*

Development of resistance to each of the 4 pseudopeptides was analyzed against various MDR clinical isolates over a 2 wk period in vitro. Serial passages were performed with each biomimetic at sub-inhibitory concentrations, concentrations below the minimal inhibitory concentrations (MICs). The pseudopeptides were compared to drugs that possess a high frequency of spontaneous resistances (rifampicin, fosfomycin) and to reference drugs for gram-positive (vancomycin) and gram-negative (colistin) pathogen treatments. After 2 weeks, the MIC of rifampicin increased about 30,000-fold for *S*. *aureus*, while the MIC of fosfomycin increased 1,000- to 2,000-fold for both *P*. *aeruginosa* and *E*. *coli* (Table 2). Over that period, the MICs of the pseudopeptides for methicillin-susceptible *S*. *aureus* (MSSA), MRSA, *E*. *coli*, and *P*. *aeruginosa* clinical strains were either unchanged or only increased a maximum of 4-fold (Table 2). We also tested resistance development on a colistin-resistant *E*. *coli* strain harboring the plasmid-mediated colistin resistance determinant MCR−1 [13]. Colistin (polymyxin E) is a “last resort” antibiotic and, like our pseudopeptides, is a cyclic peptide. After 14 d of culture in vitro, no resistance was observed in *E*. *coli* MCR−1(+) against Pep15, Pep16, and Pep19, with MICs between 8 to 16 μg/ml (Table 2). Pep18, made of natural amino acids, had the most frequent occurrence of increased MICs after serial passages, with increased resistance in all strains tested, except for *P*. *aeruginosa*. It suggests that such amino acid analogs may attenuate resistance development. We also monitored resistance after systemic or cutaneous mouse infection with MRSA treated for 4 d (severe sepsis) or 6 d (skin) with either Pep16 or Pep19. We recovered either the kidneys or the skin abscesses, and after bacterial isolation, MICs of these two compounds were determined for sets of 15 independent colonies. During that time, MRSA susceptibility to Pep16 and Pep19 was only 2- to 4-fold modified (Table 2), indicating that after systemic or local treatments against MRSA in mice, no significant resistance had developed.

**Table 2:** Assessing resistance acquisition by various gram-positive and -negative bacteria in vitro and in mice for standard antibiotics and new peptidomimetics.

					Gram-positive clinical isolates					Gram-negative clinical isolates				
		MSSA		MRSA		*P*. *aeruginosa* 15643		*E*. *coli* K12		*E*. *coli* 13090		*E*. *coli* MCR1		
	d_0_	d_14_	d_0_	d_14_	d_4_ mice with septic kidneys	d_6_ mice with skin abscess	d_0_	d_14_	d_0_	d_14_	d_0_	d_14_	d_0_	d_14_
**Pep15**	4 (4.6)	4 (4.6)	2 (2.3)	2 (2.3)	-	-	8 (9.2)	32 (36.8)	4 (4.6)	16 (18.4)	8 (9.2)	16 (18.4)	8 (9.2)	16 (18.4)
**Pep16**	4 (4.5)	4 (4.5)	4 (4.5)	4 (4.5)	4 (4.5)	8 (9)	8 (9)	16 (18)	4 (4.5)	16 (18)	8 (9)	16 (18)	8 (9)	8 (9)
**Pep18**	16 (16.4)	64 (65.6)	8 (8.2)	16 (16.4)	-	-	16 (16.4)	16 (16.4)	16 (16.4)	64 (65.6)	16 (16.4)	64 (65.6)	16 (16.4)	64 (65.6)
**Pep19**	4 (4.4)	4 (4.4)	4 (4.4)	8 (8.8)	8 (8.8)	16 (16.2)	8 (8.8)	16 (16.2)	4 (4.4)	8 (8.8)	8 (8.8)	8 (8.8)	8 (8.8)	8 (8.8)
**Vancomycin**	0.5 (0.7)	1 (1.4)	1 (1.4)	1 (1.4)	1 (1.4)	1 (1.4)			-	-	-	-	-	-
**Rifampicin**	0.03 (0.025)	1,024 (843)	1,024 (843)	1,024 (843)	-	-			-	-	-	-	-	-
**Polymyxin B**	-	-	-	-	-	-	0.5 (0.65)	1 (1.3)	0.5 (0.65)	1 (1.3)	1 (1.3)	1 (1.3)	2 (2.6)	8 (10.4)
**Colistin**									0.5 (0.65)	1 (1.3)	1 (1.3)	1 (1.3)	-	-
**Fosfomycin**	_-_	_-_	_-_	_-_	_-_	_-_	0.2 mM (0.027 g.L^−1^)	405 mM (56 g.L^−1^)	0.2 mM (0.027 g.L^−1^)	405 mM (56 g.L^−1^)	0.2 mM (0.027 g.L^−1^)	202 mM (28 g.L^−1^)	0.2 mM (0.027 g.L^−1^)	202 mM (28 g.L^−1^)

Concentrations are provided in μM and corresponding mg. L^−1^ in parentheses unless otherwise indicated. In vitro testing: 14 d serial passages with a sub-MIC of drug on MDR gram-positive and -negative pathogens; rifampicin and fosfomycin were positive controls for resistance acquisition by gram-positive and -negative bacteria, respectively. The greatest frequency of resistance acquisition occurred with Pep18, which lacks amino acid analogs. Testing resistance in infected mice: IV (approximately 5 × 10^8^ CFUs) or subcutaneous (approximately 5 × 10^9^ CFUs) MRSA injections, and subsequent treatments with 1.5 mg.kg^−1^ of Pep16, Pep19, or vancomycin. MIC assessments were done on sets of 15 independent colonies, 5 per mouse, and the results for both treatments times were similar. IV: on colonies isolated from mice kidneys, extracted 4 d after the infection, treated for a mild sepsis by a single injection at either 3 h or 15 h post infection. Subcutaneous: on colonies isolated from cutaneous abscesses from mice, extracted 6 d after the infection, treated once by IV 24 h post infection. Data associated with this Table can be found in S5 Fig. The data are representative of independent biological triplicates.

**Abbreviations:** “-,” undetermined; CFU, colony-forming unit; MIC, minimal inhibitory concentration; MRSA (Mu3), methicillin-resistant *S*. *aureus*; MSSA (Newman), methicillin-susceptible *S*. *aureus*

Overall assessment of pseudopeptide toxicity was performed on human cells, zebrafish embryos, and mice. Analyses of the effects of increasing peptide concentrations onto human kidney cells and erythrocytes were performed and directly compared to the antibacterial data (Fig 1C and 1D for Pep16 and Pep19, respectively, and S4 Fig for the other two drugs). Bactericidal concentrations of the drugs were at least approximately 50-fold lower than those starting to be toxic for human kidney cells or erythrocytes. Additional tests were performed on blood collected from treated mice (2 mg.kg^−1^, IV, single dose) to assess putative nephrotoxicity of the compounds, on sets of 3 mice per pseudopeptide (Na^+^, K^+^, Cl^−^, urea, and creatinine, S2 Table). All the recorded blood parameters assessing kidney functions were unaffected after IV treatment of each of the 4 peptides, compared to physiological values recorded on untreated mice (S2 Table). Also, 1 to 100 mM of each peptide were injected into zebrafish embryos [14]. At concentrations up to 10 mM, no pseudopeptide showed toxicity (S3 and S4 Tables). Each pseudopeptide’s potential toxicity was also assessed in mice after IV injection of single doses ranging from 1.5 to 5 mg.kg^−1^ (resulting in a calculated blood concentration of about 18.5 to 62 μM). At the lower doses, we did not see any effects on body weight or food consumption, and there were no clinical signs to indicate toxicity (S5 Table). Transient, dose-dependent, pseudo-allergic reactions (red ears and eyes, dyspnea) that started at 2.5 mg.kg^−1^ were also attenuated by drip IV (S5 Table). All recorded blood parameters were unaltered except for transient higher neutrophil counts that were seen at the highest dose of 5 mg.kg^−1^. There were fewer neutrophils when we changed the administration from bolus to a slower IV drip (S6 Table). For each peptide, the no-observed-adverse-effect level (NOAEL) for IV injection was estimated at 2 mg.kg^−1^.

### Efficacy against MRSA in systemic mouse infection models

After it was confirmed that the pseudopeptides were nontoxic in mice at therapeutically relevant and well-tolerated concentrations, we analyzed their ability to cure MRSA infections on mild and severe mouse sepsis (Fig 1E–1J), as well as in MRSA and *P*. *aeruginosa* mouse skin abscess models (Fig 1K–1M). These models mimic common systemic [15] and localized [16] human *S*. *aureus* infections, respectively, which frequently require antibiotic therapy. In vivo efficacy of peptides is usually restricted because they are subject to proteolytic degradation. Peptides are processed in mammal serum, and proteolysis is the major elimination pathway for peptide drugs [17]. However, the 4 cyclic compounds remained potent and stable in both human and mice sera and had high half-lives of 16 to 37 h after single dose incubation (S7 Table). This implies that a single-dose treatment could be effective on mild sepsis. To test this, mice were infected with MRSA at a concentration known to cause death within 7 d of the infection in about 50% of cases [18]. Either 3 h or 15 h after infection, mice had a 1.5 mg.kg^−1^ single-dose IV treatment with a peptide or vancomycin, a reference antibiotic for treating MRSA infection [19]. Statistically, the animals treated with a single dose of Pep19 survived better than those without treatment, especially when treated 15 h post infection (Fig 1F and 1G). Pep15, Pep16, or Pep18, however, had no effect during this period (S3B and S3C Fig). A severe sepsis model that kills untreated infected mice at 2 d post infection was implemented for MRSA. The results showed that peptide analogs Pep16 and Pep19, at low repeated doses (0.5 mg.kg^−1^ per day), protected the infected animals (Fig 1H and 1I), whereas vancomycin—at an identical dose—was much less effective (Fig 1J). We directly compared our in vivo data to clinical-stage drug candidate brilacidin [20], which was injected on sets of 10 infected mice at multiple doses identical to those for Pep16 and Pep19. Brilacidin cured the infected animals at doses identical to those tested for Pep16 and Pep19. The differences between brilacidin and each of the two pseudopeptides were not statistically significant, but brilacidin tended to save more infected animals. These data indicated that Pep16 and Pep19 do compare well with this clinical-stage drug candidate. We conclude from these experiments that pseudopeptides Pep16 and Pep19 are effective against MRSA on severe sepsis models. We checked whether clones with increased resistance emerged in vivo. On the severe sepsis model with MRSA, kidneys were extracted at day 7 after repeated treatments with either Pep16 or with Pep19. For treatment with Pep19, no clones with increased resistances were detected on plates containing drug at 2-fold MIC (S5 Fig), but we detected a few resistant clones when plates were treated with Pep16.

### Efficacy against MRSA or *P*. *aeruginosa* in local mouse infection model

We then challenged the antibiotic activity of Pep16 and Pep19 with another animal infection model. Because *S*. *aureus* is a leading cause of skin and soft tissue infections worldwide [21], we used a mouse model (Fig 1K) of skin and soft tissue infections [22] induced by subcutaneous inoculation with MRSA. An IV single-dose treatment (1.5 mg.kg^−1^) with Pep16 and Pep19 led to a strong reduction in skin abscess volume (Fig 1L) and in their bacterial content (Fig 1L, small colony variants [SCVs], chronically colonizing skin and survive in abscesses [23]). The verification of the presence of SCVs was assessed by monitoring their intracellular ATP levels. This implies that the IV-injected pseudopeptide diffuses into mice tissue and skin abscesses. In summary, two pseudopeptides can reduce mortality induced by severe sepsis and skin MRSA infections.

We also investigated whether the antibiotic activity of Pep16 and Pep19 could extend to gram-negative bacteria (GNB). *P*. *aeruginosa* causes a variety of infections ranging from localized skin to systemic infections. The bacterium is of concern in hospital settings [24], where it is often resistant to multiple antimicrobials. We used a mouse model of skin and soft tissue infections induced by subcutaneous inoculation with *P*. *aeruginosa* [22] (strain LESB58, MICs of 4 μM against Pep16 or Pep19, and 1 μM against colistin, performed in biological triplicates). Multiple-dose subcutaneous treatments with Pep16 or Pep19 (30 mg.kg^−1^) led to an approximately 10- or 50-fold reduction of the bacterial load (Fig 1M). Treatment with colistin at multiple doses (positive control; 9 mg.kg^−1^) also lowered the number of bacteria (Fig 1M, lower panel). We conclude from these data that Pep16 and Pep19 are active against both systemic and local MRSA infections, as well as on local infections triggered by *P*. *aeruginosa*.

### Antibiotic mechanisms of action

How do these drugs kill gram-positive and -negative human bacterial pathogens? We used scanning and transmission electron microscopy (SEM and TEM, respectively) to examine the ultrastructural changes triggered in MRSA by each peptidomimetic at the MIC (Fig 2A and S6 Fig). The treated MRSA show inflated and distorted shapes, and their cell walls seemed eroded by Pep15, Pep16, and Pep19 but not by Pep18. TEM micrographs suggest membrane deformation and detachment, with internal lateral expansion and stacks (Fig 2A). Leakage assays were performed on MSSA and MRSA. Membrane permeation was demonstrated for both MSSA and MRSA (Fig 2G and 2H), revealing that the *S*. *aureus* membranes were disrupted by each of the 4 pseudopeptides. Pep16 and Pep19 are the two most active pseudopeptides onto MRSA membranes, results that fit well with their activity on sepsis and skin mice models (Fig 1).

SEM and TEM were also examined in gram-negative cells (*E*. *coli* was used instead of *P*. *aeruginosa*, for safety concerns on the microscopes) exposed to each pseudopeptide at their respective MIC, evidencing membrane and cytoplasmic deformations, as well as cell division defects (Fig 2B). To explore their ability to permeate gram-negative outer and inner membranes, leakage assays (Fig 2C) were also conducted on *E*. *coli* cells. All pseudopeptides permeate the outer (Fig 2D) and inner (Fig 2E) *E*. *coli* membranes. Pep19 is the most active, in accordance with its activity against *P*. *aeruginosa* on a skin infection model (Fig 1N). Thus, these new drugs act by membrane permeation of GNB and gram-positive bacteria (GPB), with perturbations inside the cells.

### Structures and dynamics of two peptide analogs

**Fig 3:**
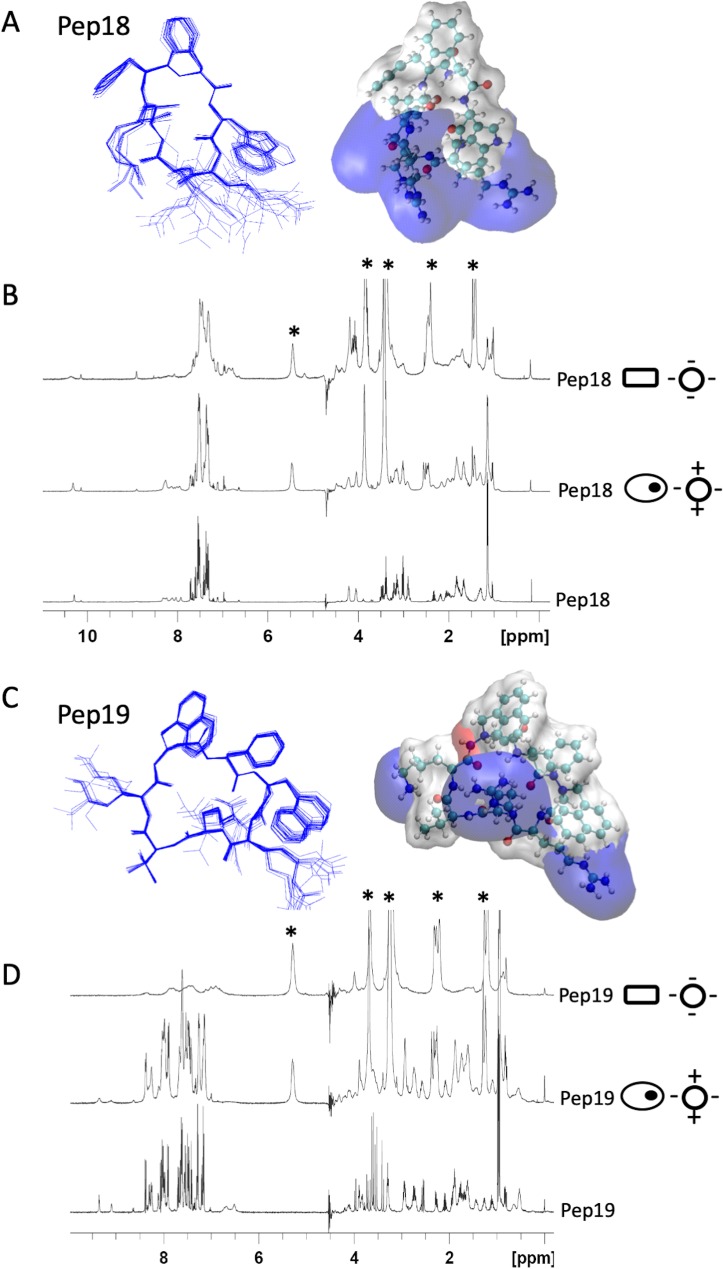
Three-dimensional structures of two pseudopeptides and their dynamics with eukaryotic and prokaryotic membrane mimics. NMR structures of Pep18 (A) and Pep19 (C) analogs in presence of SDS micelles. These differ only in the replacement of 2 phenylalanine residues in Pep18 by 2 aza-β3-1-naphthylalanyl residues in Pep19. Pep18 cyclic backbone has 7 natural amino acids. Two hydrogen bonds involve Val6 carbonyl oxygen with HN Phe2 (residue i + 2) and HN Trp3 (residue i + 3). Two aza-β3-naphthylalanine residues from Pep19 form 2 R-hydrazino turns with 2 hydrogen bonds between the amide proton of residue i with the lone pair of the sp3 nitrogen atom of residue (i − 1) and the carbonyl of residue (i − 2). One of the hydrazino turns involves residues 2–4 and is reinforced by an additional bond between the carbonyl of Phe2 and the amine of Arg5. The ternary nitrogen configuration of the aza-β3-amino acids is an R absolute configuration. The electrostatic potential surface maps of Pep18 and Pep19 in the presence of SDS micelles are depicted with blue cationic areas and white hydrophobic domains. (B, D) ^1^H NMR spectra of 2 mM peptide analogs (panel B: Pep18; panel D: Pep19) without SUV (lower spectra), in the presence of zwitterionic SUVs (middle; 8 mM DMPC-d54) or anionic SUVs (top; 8 mM DMPC-d54/DMPS-d54 70:30). Zwitterionic and anionic SUVs mimic eukaryotic (ovals with an inner black dot) and bacterial (rectangles) membranes, respectively. “*” corresponds to the lipid signals. Data associated with this figure can be found in S8–S11 Tables. Arg,arginine 5; DMPC-d54, 1,2-dimyristoyl-d54-sn-glycero-3-phosphocholine; DMPS-d54, 1,2-dimyristoyl-d54-sn-glycero-3-phosphoserine; HP-NCF, hydrolyzed product–nicrocefin; NMR, nuclear magnetic resonance; Phe2, phenylalanine 2; ppm, parts per million; SDS, sodium dodecyl sulfate; SUV, small unilamellar vesicle.

Because the peptide analogs interact with and permeabilize bacterial membranes, the atomic structures of inactive Pep18 and active Pep19 were solved by molecular modeling under nuclear magnetic resonance (NMR) constraints in a micellar environment (Fig 3A and 3C and S8–S11 Tables). Despite having only 2 distinct residues, we identified significant differences between their electrostatic potential surface maps (Fig 3). Pep18 is amphipathic and contains assembled cationic charges, whereas Pep19 has 3 independent cationic areas that form a tripod. Compared to inactive Pep18, Pep19 hydrophobic domain is extended, with 3 aromatic cycles forming π stacking interactions. These differences are relevant for the association of the pseudopeptides with the membrane through electrostatic and hydrophobic interactions. Based on the different phospholipid compositions of bacterial and eukaryotic membranes, anionic small unilamellar vesicles (SUVs) mimic bacterial membranes, whereas zwitterionic SUVs mimic eukaryotic cell membranes. For active Pep19, moderate broadening occurred in the presence of eukaryotic membrane mimics (zwitterionic SUVs) compared with the addition of negatively charged SUVs, which act as a bacterial mimic. We interpret this to suggest substantial interaction of Pep19 with the anionic SUVs mimicking bacterial membranes. By contrast, the extent of signal broadening differences induced by the presence of the zwitterionic and the anionic SUV is much less pronounced in inactive Pep18, implying weaker interactions and faster dynamics with the bacterial membrane mimic, possibly accounting for its lack of activity on infected mice. The difference in the dynamic behavior of the peptide interaction with these two SUV models supports a weaker interaction with zwitterionic membrane that could account for the lack of toxicity on eukaryotic cells but a tighter association with negatively charged SUVs, in agreement with their efficient antibacterial activity. These conclusions are also consistent with a weaker interaction of Pep18 with the negatively charged SUVs, as well as its lower antibacterial activity. The structural differences detected between Pep18 and Pep19, however, may not be related to antimicrobial potency. The naphthylalanine moieties from Pep19, however, are expected to improve antibacterial activity of peptides [25]. These bulky hydrophobic side chains are involved in π stacking interactions, reinforcing potential interactions with the membrane that could be one reason why Pep19 has activity in vivo. The reduced proteolytic stability of Pep18 (S7 Table) may also account for the lack of activity in infected animals and lower activity against bacteria.

### Conclusions

The current clinical pipeline contains around 30 new antibacterial drugs with activity against priority pathogens and is dominated by derivatives of established classes [26]. Our biomimetic approach based on toxins from microorganisms could lead to antibiotic discovery, especially according to the escalating evolution of resistance coupled with a diminished antibiotic pipeline. The main outcome of this work is that peptide analogs inspired by a substituted piece of a bacterial toxin show potential efficacy in severe systemic and local infections using animal models caused by MRSA, and 2 (Pep16 and Pep19) are active against *P*. *aeruginosa* skin infections. They do not appear to lead to resistance in MDR pathogens. We have identified therapeutic agents that can provide alternative treatments against antimicrobial resistance. The barrier to development of many membrane-acting antibiotics has been lack of efficacy in systemic infections, usually due to poor stability and/or toxicity. In this study, these obstacles were successfully passed, showing high efficacy in systemic and skin infections. A recent exception is brilacidin, a membrane-acting mimetic of a defensin AMP [27] that completed a phase II study against systemic *S*. *aureus* infections. The antibiotic activities of Pep16 and Pep19 on MRSA-infected mice (severe sepsis) was compared to that of brilacidin, and the results are promising. Both Pep16 and Pep19 are likely candidates for drug development against *S*. *aureus* sepsis, whereas Pep16 would be our lead to treat skin and soft tissue infections (Fig 1L). These novel membrane-active drugs could be used in combination with antibiotics used in the clinic, enhancing efficacy. Antibiotics targeting bacterial membranes are an underexploited mechanism for treating persistent infections [28]. It is possible that further development of this series of cyclic pseudopeptides will produce drug candidates. Pharmacodynamic profiles of these new antibiotics against MRSA should be performed in a murine thigh infection model [29]. A wider implication of our findings is that toxins should be considered as sources for new antibiotics against MDR gram-positive and -negative bacteria.

## Methods

### Ethics statement

Mice were used for this study. All experimental protocols were approved by the Adaptive Therapeutics Animal Care and Use Committee (APAFiS #4508-2016031l15541258 v2).

### Strains, media, and chemicals

We worked with *S*. *aureus* Newman, *S*. *aureus* MRSA (N315, Mu3), *E*. *coli* K12 XL1-Blue, *E*. *cloacae* (ATCC13047), *P*. *aeruginosa* (UCBPP-PA14 and LESB58), and various ESKAPE clinical isolates from Caen University Hospital (France) and the National Reference Center for Enterococci (Rennes). The growth media used were the MHB, TSB, and 2YT medium. Sigma provided the nisin, methicillin, vancomycin, daptomycin, fosfomycin, rifampicin, polymyxin B, colistin, ONPG, and DCM. Dr. DeGrado (California San Francisco University) provided Brilacidin. Also used were nitrocefin (Calbiochem); Fmoc-protected amino acids (Novabiochem and Iris Biotech); 2-chlorotrityl chloride resin (Iris Biotech); and DMF, DIPEA, HCTU, HOBt, and TFA (Biosolve Chimie).

### Peptide and pseudopeptide synthesis

The first amino acid was anchored to a CTC resin (100–200 mesh; 1.2 mmol/g, 1 g). Under nitrogen, the resin was swelled into 10 ml dry DCM for 10 min. The first monomer was attached onto the resin by adding 1.2 eq of Nα-Fmoc or Nβ-Fmoc-aza-β3-amino acids in 10 ml dry DCM and 4 eq DIPEA, then shaking for 4 h at room temperature under nitrogen. The resin was washed sequentially with DMF (5 × 10 ml) and dry DCM (3 × 10 ml). Resin was treated with DCM/MeOH/DIPEA (17:2:1; 2 × 10 ml) for 20 min and washed with DMF (3 × 10 ml). The resin loading level was determined by Fmoc release upon piperidine treatment. Cyclic pseudopeptide synthesis was performed using Nα-Fmoc-β and Nβ-Fmoc-aza-β3-amino acids [30]. Peptidomimetics were assembled on the resin by automated Fmoc synthesis on a CEM μwaves Liberty 12-channel synthesizer. Sequential synthesis of peptides was accomplished by repeated cycles of (i) deprotection with piperidine/DMF (20%); (ii) DMF washes (4 × 5 ml); (iii) coupling with 0.2 M Fmoc-α or Fmoc-aza-β3-amino acid (4 eq), 0.5 M HCTU (10 eq), and 2 M DIPEA (16 eq); (iv) and DMF washes (4 × 5 ml). Fmoc-Arg required an additional coupling step. Resin was washed with DCM, dried, and then treated with 3% TFA in DCM (3 × 10 ml) for 1 h. The resin slurry was filtered and washed with DCM, and then the cleavage solution was neutralized with N-methylmorpholine (1 eq/TFA) and diluted in DCM (170 ml). Linear peptides were added slowly to EDC (4 eq), HOBt (4 eq), and DIPEA (4 eq) in DCM (to 10−4 M). The mixture was stirred for 7 d, concentrated down to 100 ml, and then washed with 0.5 M HCl, water, and saturated NaCl. Side chain deprotection was done with TFA/H2O/TIS (95:2.5:2.5; 10 ml; 3 h). After filtration, pseudopeptides were precipitated in chilled diethyl ether, centrifuged at 4°C (3,000*g*, 10 min), and the supernatants were discarded. Peptides were analyzed by RP-HPLC in a Waters 2696 system on an XBridge C18 column (4.6 × 150 mm; 5 μm). For elution (1 ml/min flow rate) and UV detection (215 nm), we used linear 5%–60% gradients of (H2O/TFA 0.1%) over 40 min and 60%–95% gradients of (MeCN/TFA 0.1%) in 10 min. Peptides were purified by RP-HPLC on an XBridge C18 semi-prep column (19 × 1 mm; 5 μm) in a Waters 600 system to a final purity of ≥95% and lyophilized. Peptides were characterized by mass spectrometry on a microflex LT MALDI-TOF system (Bruker Daltonics). c(Ψ2-Nal-F-Ψ2-Nal-RR-ΨHyt-K), Pep15: Yield after purification 18%; white powder; RP-HPLC Rt: 28.8 min; MALDI-TOF MS mass: m/z found 1156.46 [M+H^+^]; calc.: 1156.62[M+H+]. c(Ψ2-Nal-F-Ψ2-Nal-RR-ΨV-K), Pep16: Yield after purification 21%; white powder; RP-HPLC Rt: 29.3 min; MALDI-TOF MS mass: m/z found 1226.69 [M+H^+^]; calc.: 1226.64 [M+H+]. c(FFWRRVK), Pep18: Yield after purification 20%; white powder; RP- HPLC Rt: 25.9 min; MALDI-TOF MS mass: m/z found 1020.62 [M+H^+^]; calc.: 1020.60 [M+H+]. c(Ψ1-Nal-F-Ψ1-Nal-RRVK), Pep19: Yield after purification 17%; white powder; RP- HPLC Rt: 29.1 min; MALDI-TOF MS mass: m/z found 1111.83 [M+H^+^]; calc.: 1111.63[M+H+]. As previously determined for similar compounds, the peptide and pseudopeptide concentrations for all experiments were calculated as TFA salts, which assumes the association of 1 molecule of TFA per cationic residue [10].

### Stability and antibacterial assays

Nisin, PMX, daptomycin, and pseudopeptide stabilities in human and mice sera were monitored by 1.35 ml of 25% human or mouse sera in an Eppendorf LoBind tube pre-incubated at 37°C for 15 min before adding the peptide or pseudopeptide [8] (10−4 M final concentrations). At selected time intervals, 100 μl of reaction mixture was collected, quenched by 200 μl 95% EtOH, and adjusted at pH 2 to prevent peptide adsorption to serum proteins. Mixtures were then placed at 4°C (15 min) and centrifuged at 14,000 rpm. Supernatants were analyzed by RP-HPLC on an Xbridge C18 column with a linear gradient of water, 0.08% TFA, and acetonitrile with 1% TFA. HPLC peak areas were used to calculate the percentages of intact compound remaining at the various time points. MIC and MBC values were determined in triplicate in many ESKAPE clinical isolates, including a dozen MDR *S*. *aureus*, by the broth microdilution method. Peptidomimetics and antibiotics were assayed in a concentration range of 1 to 128 μM. The bacterial inoculum (5 × 10^5^ colony-forming units [CFUs]/well) was prepared in MHB or CAMHB (150 μl). A Biotek Synergy 2 spectrophotometer was used to record the results after 18–20 h incubation at 35°C (±2°C). Time-kill assays were performed on an *S*. *aureus* MRSA 10^5^ CFU/ml inoculum and exposed to each peptide or to vancomycin at 30-fold MIC. After every hour of culture for 4 h, serial dilutions were plated on BHI plates and incubated overnight at 37°C, and then the CFUs were counted and expressed as log CFU/ml. Phosphate-buffered saline (PBS)-exposed *S*. *aureus* MRSA was included as a control.

### Permeability assays for gram-positive and -negative bacteria

Outer and inner membrane permeability was determined on *E*. *coli* ML-35p, a lactose permease-deficient strain [8]. Permeation assays were carried out on 100 μl MHB from bacteria cultures grown to 0.15 OD600 (10^7^ CFU/ml). Nitrocefin (20 μg/ml) was added to the tested molecules at a concentration near to their MICs, and cleavage was monitored by light absorption at 500 nm (outer membrane monitoring). Inner membrane monitoring of cleavage of ONPG (100 μg/ml) and substrate was monitored at 420 nm. Polymyxin B was a positive control, while a bacterial suspension in MHB was the negative control. Permeation assays were also performed on gram-positive *S*. *aureus* membranes (MSSA and MRSA) by fluorescence assay [31] with SYTO Green nucleic acid stain (Life Technologies) in black flat-bottom 96-well microtiter plates (Greiner) in biological triplicate. Bacteria were grown to 0.3–0.4 OD600 in CAMHB at 37°C and centrifuged at 3,500 tr/min for 10 min. The pellet was washed and dissolved in PBS to an absorbance of 0.4 at 600 nm (approximately 2.10^8^ CFU/ml). SYTO Green was added to the cells at a 5 μM final concentration, with dark incubation for 30 min. The amount of 50 μL of bacteria/SYTO Green mixture was added to each well containing compounds serially diluted in PBS, and fluorescence was measured at 37°C for up to 12 h using a spectrophotometer (BIOTEK, Tungsten-Halogen Lamp) with 485 nm excitation and 525 nm emission wavelengths.

### Electron microscopy

*S*. *aureus* Newman and *E*. *coli* K12 were cultured in MHB to an OD600 of 0.5 (10^8^ CFU/ml). After adding peptides, samples were incubated at 160 rpm for 2 h at 37°C before centrifugation. Bacteria were exposed to pseudopeptide concentrations at their MIC. Untreated controls were prepared under identical conditions. Cell pellets were obtained after cell suspension centrifugation (1,000–3,000*g* for 5 min). Samples were fixed for 20 h with 2.5% glutaraldehyde in 0.1 M cacodylate buffer (pH 7.2), rinsed, fixed again for 2 h with 1% OsO4 in the same buffer, and washed. *S*. *aureus* Newman samples were embedded in 2% agar and dehydrated in a graded series of increasing ethanol concentrations (50, 70, 90, and 100% v/v). For *E*. *coli* K12, the samples were placed on a 0.2 μm filter disc, dehydrated in a series of increasing ethanol concentrations, and vacuum dried for 48 h. For SEM observation, specimens were obtained after preparation with a Leica EM CPD300 critical point dryer. A Leica EM ACE200 was used for gold palladium metallization for 30 s. Sample observation was performed with a JEOL JSM-6301F microscope at 7 kV with a 15 mm work distance. TEM was also used to visualize the morphology and intracellular alteration of peptide-treated bacterial cells. For this, bacteria were grown and incubated with AMPs as described above for SEM sample preparation, and controls were prepared in standard medium. Cell pellets were also prepared as described above. The same buffers and dehydration protocols were used, but this time they were followed by a graded acetone series and then embedding in epoxy resin (Polysciences Embed-It low-viscosity epoxy kit). Ultrathin sections were prepared on Formvar-coated grids (Plano) and stained with 3% uranyl acetate. Microscopy was performed with a 120-keV Zeiss 912 Omega (Oberkochen) microscope. To optimize the contrast, zero-loss energy filtering was applied.

### Resistance studies

Vancomycin, polymyxin B, fosfomycin, rifampicin, and pseudopeptide MICs were determined for *S*. *aureus* Newman, MRSA, *P*. *aeruginosa* 15643, and LESB58, as well as *E*. *coli* K12 and 13090. For 15 d in MHB, MICs were determined daily for each pseudopeptide and their antibiotic controls. This was done using cells from the well in which the compound concentration was one-half of the MIC (1/2MIC). For each compound, the 1/2MIC well from the previous day’s MIC assay plate was re-suspended and the bacteria grown to a density of about 10^8^ cells ml−1. The suspension was diluted in MHB or CAMHB (10^6^ cells/ml) and then used to determine the MIC of the same compound determined by micro broth dilution [32]. All MIC measurements were performed in duplicate. Aliquots of the 5 × 10^4^ cell/ml dilutions for each 1/2MIC well were periodically plated on MHB and incubated at 35°C for 18 h to verify cell density by colony counts and for visual inspection of colony phenotypes. To control compound stability, peptide solution MICs were determined during and at the end of the 15 d and were compared to cells unexposed to antibiotics. In vivo resistance was also assessed in treated mice (1.5 mg.kg^−1^) for 4 d (sepsis) or 6 d (skin abscesses). The MICs of Pep16, Pep19, and vancomycin were determined for colonies observed on BHI plates after the homogenization of excised mice kidneys 4 d after inoculation (sepsis model) or of excised abscesses 6 d afterwards (skin infection model). The broth microdilution method was performed as per EUCAST recommendations on 5 independent colonies per mouse (3 mice per set, 15 colonies per drug tested). For severe sepsis and subcutaneous abscesses, in vivo resistance was determined by plating aliquots of crude kidney extracts or abscesses on plates containing 1-, 2-, 4-, or 8-fold the peptide MICs 7 or 3 d post infections, respectively.

### Animal efficacy studies

Tolerance and toxicity studies were done on male Swiss mice (CD-1 Crl: CD-1 [ICR] IGS; Charles River). These were 7 to 10 weeks old and weighed an average of 37.2 g. All experimental protocols were approved by the Adaptive Therapeutics Animal Care and Use Committee (APAFiS #4508-2016031l15541258 v2). For the sepsis model, we used female Swiss mice (Janvier Labs), 6 to 8 weeks old and weighing approximately 30 g. Experiments were monitored in the ARCHE-BIOSIT animal lab in Rennes, in biological duplicate. We used Groups of 5 mice for the mild and the severe sepsis models with MRSA. Mice were IV inoculated with 150 μl of MRSA suspensions in 0.9% NaCl containing approximately 5 × 10^8^ CFUs for the “mild” sepsis and approximately 2 × 10^9^ CFUs for the “severe” sepsis, respectively. The infected mice received an IV dose of Pep16, Pep19, or vancomycin (1.5 mg.kg^−1^) either 3 or 15 h post inoculation for the “mild” sepsis model, or 4 IV doses (0.5 mg.kg^−1^) of either Pep16, Pep19, brilacidin, or vancomycin 2 h, 24 h, 48 h, and 72 h post infections for the “severe” sepsis model. Mice survival was monitored for 4 d (“severe” sepsis) or 14 d (“mild” sepsis), and the statistical significance of difference(s) between the groups was evaluated using the Wilcoxon test. *P* < 0.05 was considered significant. Two weeks (“mild” sepsis) or 4 d (“severe” sepsis) after inoculation, mice were euthanized with CO2.

For the cutaneous abscess model, the mice were randomly divided into 5 sets. The mice were anesthetized with isoflurane, their dorsal fur shaved, and their skin disinfected with 70% ethanol. An overnight culture of *S*. *aureus* MRSA or *P*. *aeruginosa* LESB58 was washed with 0.9% NaCl, and then a subcutaneous injection of 2 × 10^9^ CFUs and an equal amount of sterile Cytodex microcarrier beads (20 mg.ml^−1^, Sigma) for *S*. *aureus* MRSA, or 10^8^ CFUs for *P*. *aeruginosa* were injected into the shaved area. Mice were treated with Pep16, Pep19, or vancomycin (1.5 mg.kg^−1^) in the tail vein 24 h post inoculation for the MRSA subcutaneous abscess model or subcutaneously with either Pep16 (30 mg.kg^−1^), Pep19 (30 mg.kg^−1^), or colistin (9 mg.kg^−1^) directly within the abscess area twice in a day for 2 d for the *Pseudomonas* abscess model. Mice were monitored every day and then euthanized with CO2 on Day 6 or on Day 3. Abscesses were excised, measured, and homogenized in PBS using an Ultra-Turax. Serial dilutions of the samples were plated on BHI (MRSA) or 2YT medium (LESB58) plates and then incubated overnight at 37°C. Afterward, the CFUs were counted and expressed as log CFU/ml. Dermonecrosis areas were calculated according to the formula π × L × W. ATP levels were determined with an ATP assay kit (Promega) according to manufacturer instructions on sets of 5 colonies harboring the SCV phenotype, and sets of 5 colonies harboring a normal phenotype, after growth on BHI plates.

### Zebrafish embryo, mice, and human cell toxicity assays

Adult fish were kept in 30l aquariums at 27°C ± 2°C under constant light/dark (14:10 h) cycles. The day before the embryos were used, males and females were placed in breeding tanks. The fish were left undisturbed overnight, and the eggs were collected 1 h after the light was turned on the next morning. When the injections of the cyclic peptides started, embryos were transferred into microtiter plates placed at 28°C for 24 h. Peptides were prepared at 1, 10, 20, 40, and 100 mM concentrations in water, and injections were performed into the caudal vein. Each peptide was injected at 1 nl for all concentrations except 100 mM, when 2 nl was injected. Control embryos were injected with 1 nl of water. When the peptides were injected, analysis determined embryo sizes, curvatures, and cardiac frequencies. Peptide concentrations were checked in 10 embryos, and these were divided among 96-well plates and analyzed under a microscope or using a magnifying glass. Analysis was done for 9 qualitative endpoints in order to determine mortality rates and the frequencies of toxic effects, which include cardiac bleeding, blood flow defects, cerebellar bleeding, necrosis, pericardia edema, otic vesicle, and motility and brain defects. To determine the NOAELs for each drug, toxicity in mice was monitored by single IV administration (bolus or over a 30 s period) of Pep15, Pep16, Pep18, and Pep19. To do this, each peptide was prepared in 0.9% NaCl and injected into the tail veins of 3 male Swiss mice at 1.5, 2.0, 2.5, or 5.0 mg.kg^−1^, corresponding, respectively, to calculated blood concentrations of approximately 18.5, 24.5, 31, and 62.0 μM. Each animal was checked for mortality twice per day. Clinical signs were recorded daily, including any reduced motor activity, piloerection, and redness in the ear lobe, cyanosis, protruding eyeballs, slow or labored breathing, loss of response in the rear legs, convulsions, or death. Body weight was recorded at least once before the beginning of the treatment period and again on the day of treatment, with the satellite group checked once during the observation period. For the groups of mice observed for 7 d, food consumption was measured once during the study. All the animals were euthanized on the day after treatment, except for 4 satellite animal groups that were euthanized after a 7 d observation period. All then underwent complete macroscopic and microscopic post-mortem examinations. Hematology investigations were performed before necropsy for all animals except the satellite ones, whose blood biochemistry parameters were determined before euthanasia. For all injected doses, measurements were done 2 d before necropsy of various hematology parameters. These included white blood cells counts, erythrocytes counts, hemoglobin concentration, hematocrit, mean corpuscular volume, mean corpuscular hemoglobin, mean cell volume, packed cell volume, mean cell hemoglobin concentration, mean cell hemoglobin, thrombocytes, leucocytes, reticulocytes, neutrophils, eosinophils, basophils, lymphocytes, large unstained cells, monocytes, prothrombin time, fibrinogen, activated partial thromboplastin time, potassium, and creatinine. After euthanasia, selected organs were weighed, and selected tissue was preserved for microscopic examination. The human erythrocyte toxicity assays were performed as described by Solecki and colleagues [8]. Human blood was collected from the Rennes Etablissement Français du Sang. Red blood cells (RBCs) were centrifuged at room temperature to 1,400 rpm for 10 min. Supernatant was discarded and RBCs recovered into 1X PBS buffer. RBCs were centrifuged again, and the pellets resuspended in 1X PBS. RBCs were washed until the supernatant was clear. Of that suspension, 75 μl was mixed with 75 μl of 2-fold serial dilutions of purified peptide in a microtiter plate, from 0.5 to 512 μM in 1X PBS. The plates were incubated for 2 h at 37°C. After plate centrifugation (15 min, 1,400 rpm), the absorbency of the supernatant was measured at 414 nm. A solution of water 1% Triton X-100 was a positive control with all RBCs lysed, and the hemoglobin release was set at 100%. RBCs in PBS were a negative control without hemolysis. For each peptide, RBCs from 3 different donors were tested in triplicates. For the human kidney toxicity assays, HEK-293 cells were cultivated on DMEM medium supplemented with 10% fetal calf serum, 2 mM glutamine, 100 IU/mL penicillin, 100 μg/mL streptomycin, and 1% non-essential amino acids at 37°C with 5% CO2. Cells were seeded in 96-well microplates and incubated 24 h prior to treatment. HEK-293 cells were exposed to Pep 15, Pep 16, Pep 18, or Pep 19 in a 0.5 to 512 μM concentration range. After 24 h exposures, the various peptides were removed and cells incubated with NucBlue and 1 μg/mL propidium iodide for 30 min. Cells were washed in PBS, and images were taken on living cells using an automated ImageXpress Micro XLS microscope, with a 10X objective. Four fields per well have been acquired and analyzed individually using Columbus software. The IC50 of each of the 4 pseudopeptides were calculated using GraphPad Prism software.

### NMR in presence of SUVs

The phospholipids 1,2-dimyristoyl-d54-sn-glycero-3-phosphocholine (DMPC-d54) and 1,2-dimyristoyl-d54-sn-glycero-3-phosphoserine (DMPS-d54) were purchased from Avanti Polar Lipids (Alabaster, AL). Lipids were used without further purification. SUVs were prepared as described [33]. Lipids were mixed in chloroform and dried under vacuum to obtain a thin film. Addition of water formed multilamellar vesicles. After sonication of the MLV for a few minutes at room temperature, using the microtip of a sonicator (U200S, UKA Labortechnic), SUVs were obtained. Titanium debris from the sonicator probe were eliminated by centrifugation. The amount of 2 mM of Pep18 or Pep19 was dissolved in H_2_0/D_2_0 (90/10) at pH 5.5. SUVs composed of DMPC-d54 or a mixture of DMPC-d54/DMPS-d54 (70/30) were added at 200 mM. SDS d-25 was from Cortecnet (Voisins le Bretonneux, France).

### NMR and molecular modeling in presence of micelles

The NMR samples contained 1–4 mM of Pep18 and Pep19 dissolved in water or in the presence of SDS micelles (100–200 mM) at pH 5.1–5.7. The peptide/micelles ratio was 1:25 and 1:50 for P18 and P19, respectively. All spectra were recorded on a Bruker Avance 500 spectrometer equipped with a 5 mm TCI CryoProbe (^1^H, ^13^C, ^15^N). Homonuclear 2D DQF-COSY, TOCSY, and NOESY spectra were recorded using standard Bruker sequences in the phase-sensitive mode and using the States-TPPI method. NOESY spectra were acquired with 8 scans, with the resulting spectra summed in order to suppress t1 noise as recently suggested [34]. For the NOESY spectra, a mixing time of 150 ms was used. Spectra were acquired using matrices of 4,096 × 320–600 zero filled in F1 to 2K × 1K after apodization with shifted sine-square multiplication in both domains. Bruker TopSpin software was used to process the spectra, which were analyzed with NMRView [35].

### Structure calculations

^1^H chemical shifts were assigned according to classic sequential assignment procedures [36]. NOE cross-peaks were integrated and assigned within the NMRView software [35]. NOE peak volumes between methylene pair protons were used as reference of 1.8 Å. The lower bound for all restraints was fixed at 1.8 Å and the upper bounds at 2.7, 3.3, and 5.0 Å, respectively, for strong, medium, and weak correlations. Structure calculations were performed with AMBER 17 [37] in 2 parts. The initial cooking stage was performed at 1,000 K to generate 100 initial random structures. Simulated annealing calculations were then done during 20 ps (20,000 steps of 1 fs). To do this, the temperature was first increased quickly and kept at 1,000 K for the first 5,000 steps. The system was gradually cooled down to 100 K from step 5,001 to 18,000. The temperature was then brought to 0 K during the remaining 2,000 steps. For the first 3,000 steps, the force constant of the distance restraints was increased gradually from 2.0 to 20 kcal.mol−1.Å. For the rest of the simulation (steps 3,001 to 20,000), the force constant was kept at 20 kcal.mol−1.Å. The 20 lowest energy structures with no violations > 0.3 Å were representative of the compound structure. The representation and quantitative analysis were done using either MOLMOL [38] or YASARA [39]. For electrostatic comparison of the peptides, the surface potentials were computed for each of the lowest energy structures. In the case of Pep19, the aza-naphthalene group required charge fitting to the Amber03 force field, and this was done using YASARA’s standardized RESP protocol [39]. Related naphthalene parameters were added to the PDB2PQR software [40]. APBS software was used to compute electrostatic 3D maps [41] for an ionic strength of 50 mM NaCl, and these were visualized as isosurfaces using VMD software [42].

## Supporting information

S1 Fig Sequences and chemical structures of 4 cyclic peptides and pseudopeptides. Chemical structures and sequences of Pep15, Pep16, Pep18, and Pep19. Pep18 is the only one that is made entirely of natural amino acids.
(DOCX)

S2 Fig Antibiograms of 9 clinical isolates included in the MIC assay showing that the tested *P*. *aeruginosa*, *K*. *pneumonia*, and *A*. *baumanii* bacteria are MDR bacteria. AKN, amikacin; AMP, ampicillin; AN, nalidixic acid; ATM, aztreonam; CAZ, ceftazidime; CIP, ciprofloxacin; CST, colistin; CTX, cefotaxime; CZD, ceftazidin; FEP, cefepime; FOS, fosfomycin; GMN, gentamycin; IPM, imipenem; LVX, levofloxacin; MEM, meropenem; OFX, ofloxacin; PIL, piperacillin with tazobactam; SXT, Co-trimoxazole; TIC, ticarcillin; TMN, tobramycin.
(DOCX)

S3 Fig Pep15, Pep16 and Pep18 antibiotic activity. (A) Kill curves of MRSA for Pep15 (orange), Pep18 (purple), and vancomycin (blue) compared to untreated growth (black). Incubation of MRSA with 30-fold MIC. Results and error bars are representative of 3 independent experiments. (B, C) Kaplan-Meier survival probability plots of 6- to 8-week-old mice IV inoculated with approximately 5×10^8^ MRSA and monitored daily for 2 wk. Single-dose IV treatments were done on a septicemia model of MRSA either 3 h (plain lines; B) or 15 h (dotted lines; C) post infection. Treatment was with 1.5 mg.kg^−1^ Pep15 (orange), Pep16 (red), or Pep18 (purple). Survival was monitored for 14 d (Days, x-axis) after infection. The results are representative of independent experiments, with 10 mice per assay. Data associated with this figure can be found in S1 Data. MIC, minimal inhibitory concentration; MRSA, methicillin-resistant *S*. *aureus*.
(DOCX)

S4 Fig Pep15 and Pep18 antibiotic activity compared to their human erythrocyte and kidney cell toxicity. Antibacterial activity against MRSA is in red, human erythrocyte lysis is in blue bars, and HEK cell viability is in green. Means and standard errors of the means calculated on biological triplicates. Data associated with this figure can be found in S1 Data. HEK, human embryonic kidney cells; MRSA, methicillin-resistant *S*. *aureus*.
(DOCX)

S5 Fig No detectable MRSA clones with increased resistance to Pep19 emerged after the severe sepsis experiments on mice. Crude kidney extracts (a 10^5^ dilution of the crude extract is used) of 3 mice were plated after 4 d of repeated treatments with Pep19. Mice are euthanatized at Day 7 and their kidneys extracted. MRSA, methicillin-resistant *S*. *aureus*.
(DOCX)

S6 Fig Peptide and pseudopeptide bacterial damage revealed by TEM and SEM. TEM (left) and SEM (right) wide field-of-view micrographs of *S*. *aureus* Newman. Shown are untreated bacteria (Control) and bacteria after treatment with Pep15, Pep16, Pep18, or Pep19 at their MICs for 2 h at 37°C. MIC, minimal inhibitory concentration; MRSA, methicillin-resistant *S*. *aureus*; SEM, scanning electron microscopy; TEM, transmission electron microscopy.
(DOCX)

S1 Table Antibacterial activity of the 4 peptidomimetics focusing on 9 *S*. *aureus* MDR clinical isolates. Clinical isolates BCB/POE and 740404 DUN are from human sepsis. The other strains are from catheter infections or suppurating wounds. Brilacidin was used as a control. MDR, multidrug resistant.
(DOCX)

S2 Table Blood parameters assessing putative nephrotoxicity of each of the 4 peptide/pseudopeptides in mice. The blood parameters were analyzed on Day 2 after IV administration of the peptides and pseudopeptides at 2 mg.kg^−1^. The mean was inferred from the measured values for 3 mice (males) per peptide/pseudopeptide. Normal values are 145–160 mmol/L for Na^+^, 4–7.5 mmol/L for K^+^, 110–120 mmol/L for Cl^−^, 1.2–2.8 mmol/L for inorganic phosphorus, 9.5–11 mmol/L for glucose, 9–12 mmol/L for urea, and 6–14 μmol/L for creatinine (http://www.jax.org/phenome).
(DOCX)

S3 Table Qualitative toxicity assays of the 4 peptides on zebrafish embryos. Within a concentration range of 1 to 100 mM, 9 endpoints were analyzed to determine mortality rates and the frequency of various toxic effects (cardiac hemorrhage, blood flow defects, cerebellar hemorrhage, necrosis, pericardia edema, otic vesicle defects, motility defects, and brain defects). No toxicity was observed after injection of each of the 4 peptides and pseudopeptides at 1 or 10 mM concentrations, nor for those injected with Nisin or just water. However, 100% mortality was observed after injection of 100 mM of any of the peptides or pseudopeptides. CP, cumulative percentage of toxic defects; F, frequency; *N*, number of treated embryos; P, percentage.
(DOCX)

S4 Table Quantitative toxicity assays of the 4 peptides on zebrafish embryos. Three endpoints were analyzed to determine the embryo development effects of peptides injected in concentrations ranging from 1 to 100 mM. Embryo body sizes and curvatures were calculated, and no toxic effects were detected. Cardiac frequency defects were observed after injection of 20 and 40 mM Pep16, and to a lesser extent with 20 mM of Pep18 and Pep19. *N*, number of embryos alive after treatment; p15, pseudopeptide Pep15; p16, Pep16; p18, Pep18; p19, Pep19; SD, standard deviation; SEM, standard error of the mean.
(DOCX)

S5 Table Mice toxicity assays. Clinical signs observed on Day 1 after IV administration of each of the peptide and pseudopeptides in sets of 3 mice. Not observed symptoms are marked with a dash.
(DOCX)

S6 Table Blood parameters in the mice toxicity assays. White blood cell and neutrophil parameters on Day 2 after IV administration of the peptides and pseudopeptides at doses ranging from 1.5 to 5 mg/kg (3 mice per dose). g/L, giga/l (10^3^ elements per mm^3^). Normal values for white blood cells are 0.68–10.3 g/L, and 0.15–1.5 g/L for neutrophils.
(DOCX)

S7 Table Peptide half-lives in human and mouse sera. Degradation kinetics of the 4 cyclic peptides in human or mouse sera (peptide concentration: 10^−4^ M) and compared to 3 peptide reference antibiotics (nisin, polymyxin B, and daptomycin). Peptide amounts and half-lives were determined by HPLC. Experiments were conducted on 25% human or mouse sera. Values are shown in minutes. The mean values correspond to biological triplicates. HPLC, high pressure liquid chromatography.
(DOCX)

S8 Table Pep18 assignment in the presence of 100 mM SDS (H_2_O:D_2_O 90:10) at 318 K and pH 5.8. (DOCX)

S9 Table Pep19 assignment in the presence of 100 mM SDS (H_2_O:D_2_O 90:10) at 323 K and pH 5.3. (DOCX)

S10 Table Structural statistics for the 20 best models of Pep18 and Pep19 in the presence of SDS micelles. SDS, sodium dodecyl sulfate.
(DOCX)

S11 Table Torsion angles of Pep19 in the presence of SDS micelles. SDS, sodium dodecyl sulfate.
(DOCX)

S1 Data Spreadsheet that contains all the individual values that were used to create Figure panels 1B-D, 1F-J, 1L-M, 2D-E, 2GH, S3A-C and S4, for readers to assess our analysis. (XLSX)

## Abbreviations

AMP: antimicrobial peptide
CAMHB: cation-adjusted Mueller-Hinton broth
CFU: colony-forming unit
CTC: 2-chlorotrityl chloride
DCM: dichloromethane
DIPEA: N,N-diisopropylethylamine
DMF: N,N-dimethylformamide
DMPC-d54: 1,2-dimyristoyl-d54-sn-glycero-3-phosphocholine
DMPS-d54: 1,2-dimyristoyl-d54-sn-glycero-3-phosphoserine
ESKAPE: *Enterococcus faecium*, *Staphylococcus aureus*, *Klebsiella pneumoniae*, *Acinetobacter baumannii*, *Pseudomonas aeruginosa*, and *Enterobacter* spp
Fmoc: 9-fluorenylmethoxycarbonyl
GNB: gram-negative bacteria
GPB: gram-positive bacteria
HCTU: O-(1H-6-Chlorobenzotriazole-1-yl)-1,1,3,3-tetramethyluronium hexafluorophosphate
HOBt: 1-Hydroxybenzotriazole
HPLC: high-performance liquid chromatography
MALDI-TOF: matrix-assisted laser desorption/ionization time of flight
MBC: minimal bactericidal concentration
MDR: multidrug resistant
MHB: Mueller-Hinton broth
MIC: minimal inhibitory concentration
MRSA: methicillin-resistant *Staphylococcus aureus*
MSSA: methicillin-susceptible *S*. *aureus*
NMR: nuclear magnetic resonance
NOAEL: no-observed-adverse-effect level
ONPG: O-nitrophenyl-β-D-galactopyranoside
PBS: phosphate-buffered saline
PMX: polymyxin B sulfate
RBC: red blood cell
SCV: small colony variant
SDS: sodium dodecyl sulfate
SEM: scanning electron microscopy
SG: sytox green
SPPS: solid-phase peptide synthesis
SUV: small unilamellar vesicle
TEM: transmission electron microscopy
TIS: triisopropylsilane
TFA: trifluoroacetic acid
