# Supplementary material for: Novel antibiotics effective against gram-positive and -negative multi-resistant bacteria with limited resistance
Source: PLoS Biol. 2019 Jul 9;17(7):e3000337. doi: 10.1371/journal.pbio.3000337 (PMC6615598; doi:10.1371/journal.pbio.3000337)
Supplement: S1 Fig — Chemical structures and sequences of Pep15, Pep16, Pep18, and Pep19. Pep18 is the only one that is made entirely of natural amino acids. (DOCX) [file pbio.3000337.s001.docx]

| **Cyclic**  **peptides** | **Sequences** |
| --- | --- |
| Pep15 | c(Ψ2-Nal-F-Ψ2-Nal-RR-ΨHyt-K) |
| Pep16 | c(Ψ2-Nal-F-Ψ2-Nal-RR-ΨV-K) |
| Pep18 | c(FFWRRVK) |
| Pep19 | c(Ψ1-Nal-F-Ψ1-Nal-RRVK) |
