# Supplementary material for: Novel antibiotics effective against gram-positive and -negative multi-resistant bacteria with limited resistance
Source: PLoS Biol. 2019 Jul 9;17(7):e3000337. doi: 10.1371/journal.pbio.3000337 (PMC6615598; doi:10.1371/journal.pbio.3000337)
Supplement: S2 Fig — AKN, amikacin; AMP, ampicillin; AN, nalidixic acid; ATM, aztreonam; CAZ, ceftazidime; CIP, ciprofloxacin; CST, colistin; CTX, cefotaxime; CZD, ceftazidin; FEP, cefepime; FOS, fosfomycin; GMN, gentamycin; IPM, imipenem; LVX, levofloxacin; MEM, meropenem; OFX, ofloxacin; PIL, piperacillin with tazobactam; SXT, Co-trimoxazole; TIC, ticarcillin; TMN, tobramycin. (DOCX) [file pbio.3000337.s002.docx]

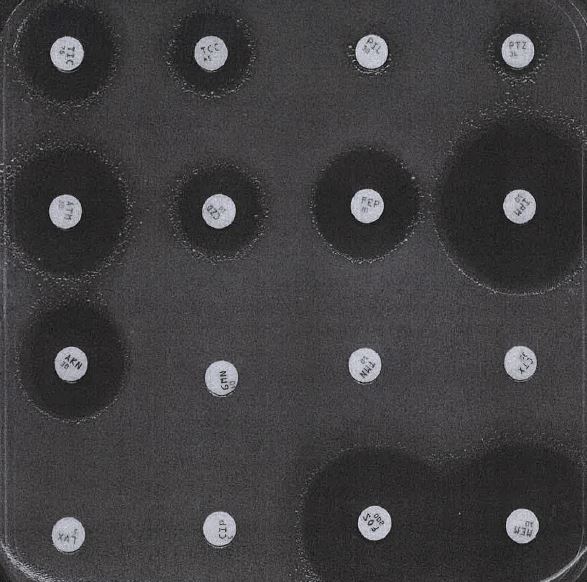

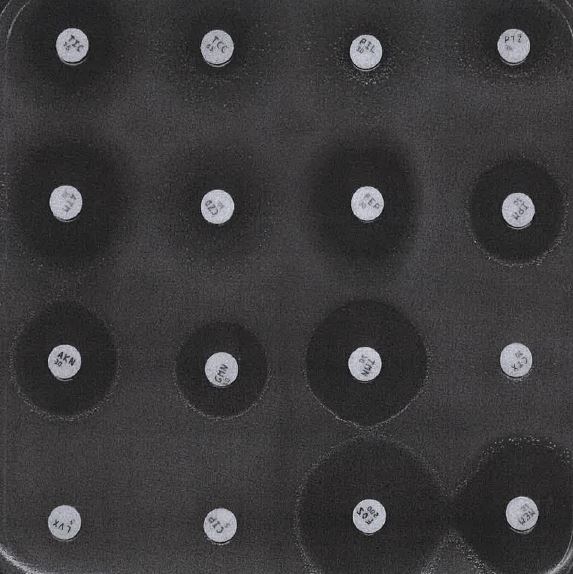


***P. aeruginosa* 15637 *P. aeruginosa* 15638**


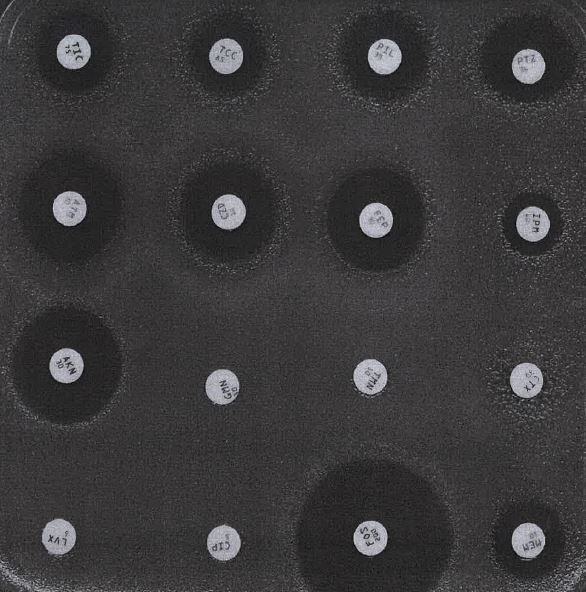

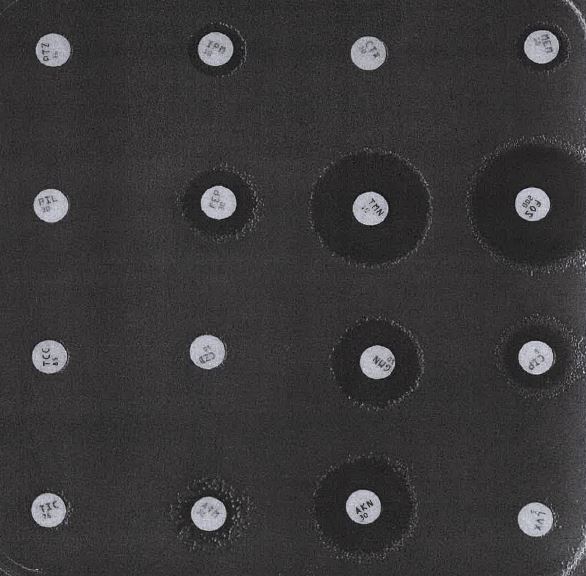


***P. aeruginosa* 15641 *P. aeruginosa* 15643**


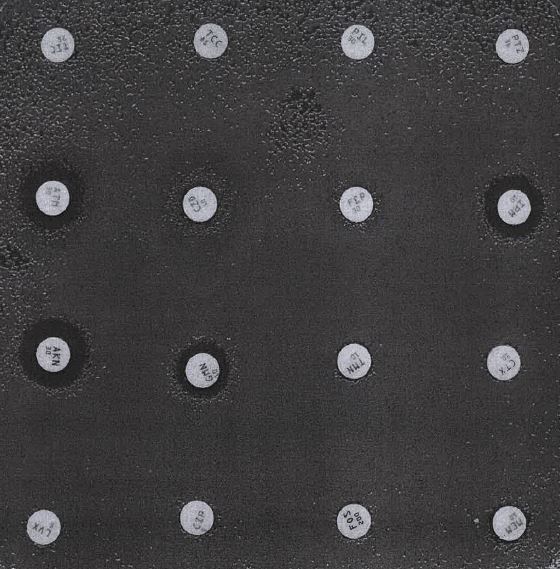


***P. aeruginosa* 15644**


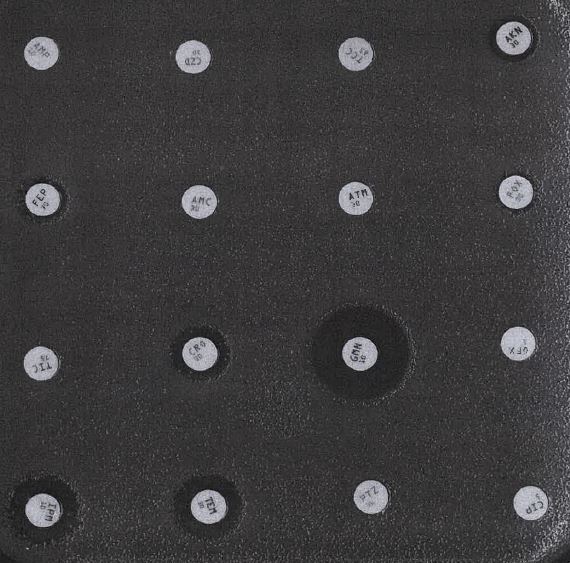

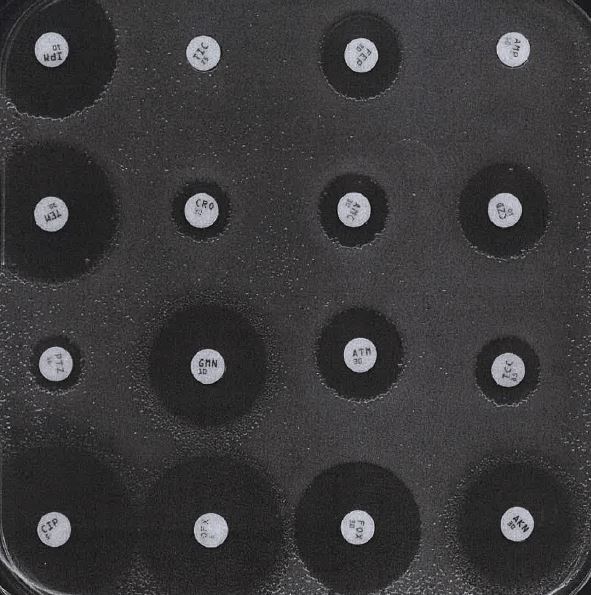


***K. pneumoniae* 13825 *K. pneumoniae* 13829**


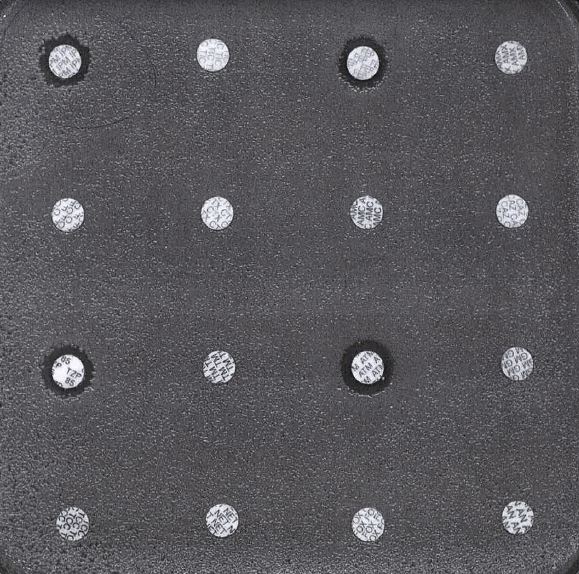

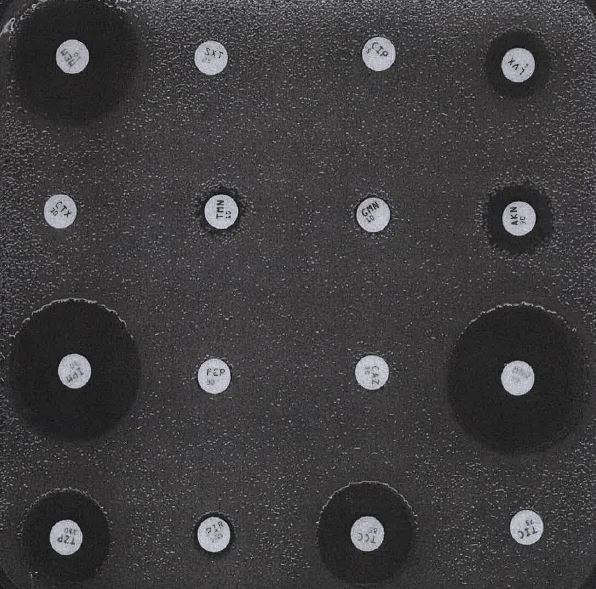


***A. baumanii* 11896 *A. baumanii* 7985**
