# Supplementary material for: Novel antibiotics effective against gram-positive and -negative multi-resistant bacteria with limited resistance
Source: PLoS Biol. 2019 Jul 9;17(7):e3000337. doi: 10.1371/journal.pbio.3000337 (PMC6615598; doi:10.1371/journal.pbio.3000337)
Supplement: S3 Fig — (A) Kill curves of MRSA for Pep15 (orange), Pep18 (purple), and vancomycin (blue) compared to untreated growth (black). Incubation of MRSA with 30-fold MIC. Results and error bars are representative of 3 independent experiments. (B, C) Kaplan-Meier survival probability plots of 6- to 8-week-old mice IV inoculated with approximately 5×10^8^ MRSA and monitored daily for 2 wk. Single-dose IV treatments were done on a septicemia model of MRSA either 3 h (plain lines; B) or 15 h (dotted lines; C) post infection. Treatment was with 1.5 mg.kg−1 Pep15 (orange), Pep16 (red), or Pep18 (purple). Survival was monitored for 14 d (Days, x-axis) after infection. The results are representative of independent experiments, with 10 mice per assay. Data associated with this figure can be found in S1 Data. MIC, minimal inhibitory concentration; MRSA, methicillin-resistant S. aureus. (DOCX) [file pbio.3000337.s003.docx]

A.


B. C.
