# Supplementary figures and images for: Novel antibiotics effective against gram-positive and -negative multi-resistant bacteria with limited resistance
Source: PLoS Biol. 2019 Jul 9;17(7):e3000337. doi: 10.1371/journal.pbio.3000337 (PMC6615598; doi:10.1371/journal.pbio.3000337)

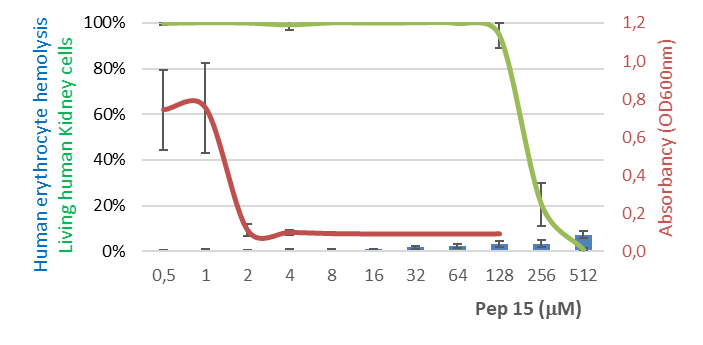

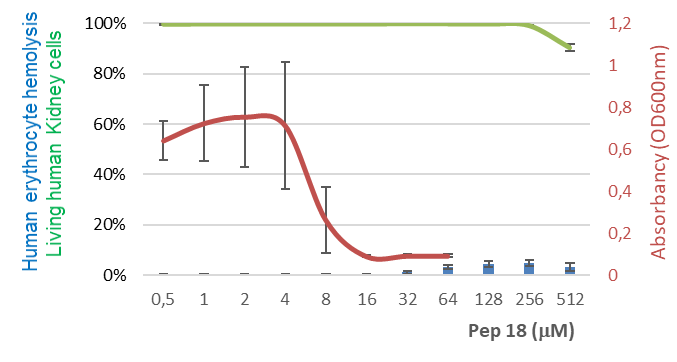

Supplement: S4 Fig — Antibacterial activity against MRSA is in red, human erythrocyte lysis is in blue bars, and HEK cell viability is in green. Means and standard errors of the means calculated on biological triplicates. Data associated with this figure can be found in S1 Data. HEK, human embryonic kidney cells; MRSA, methicillin-resistant S. aureus. (DOCX) [file pbio.3000337.s004.docx]

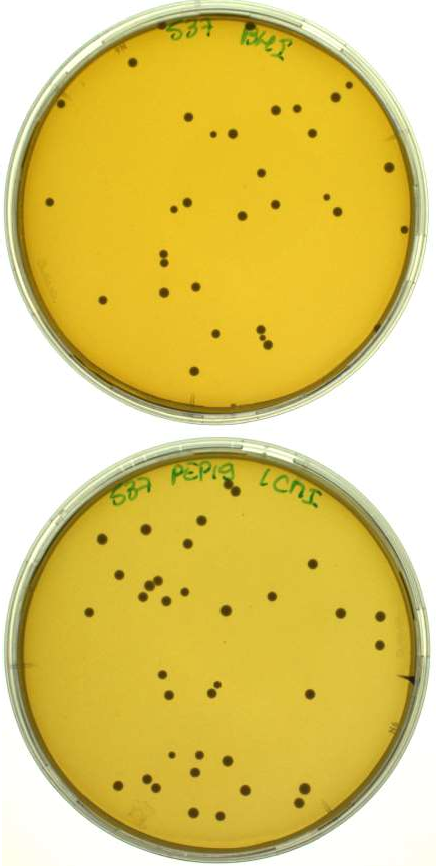

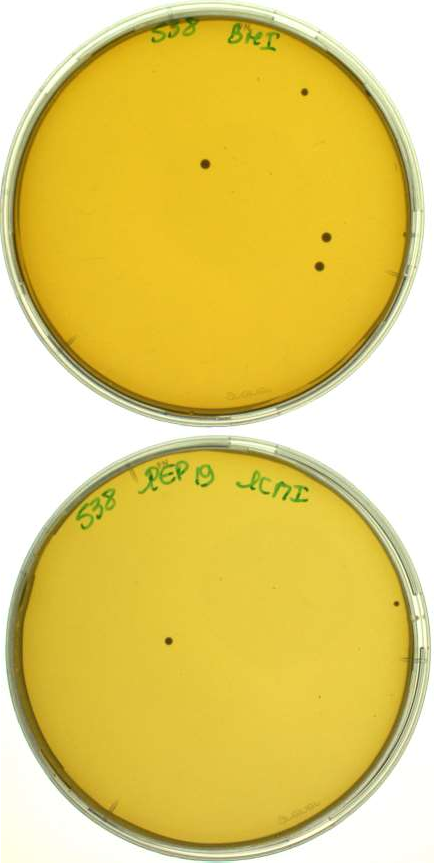

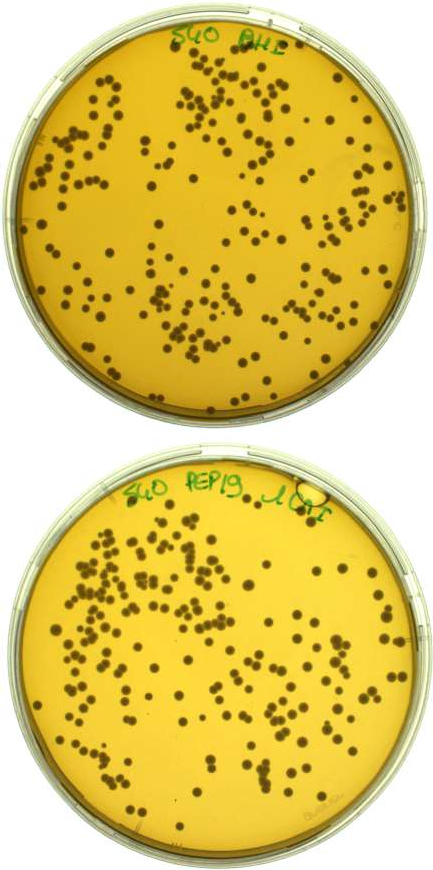

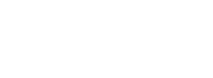

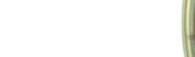

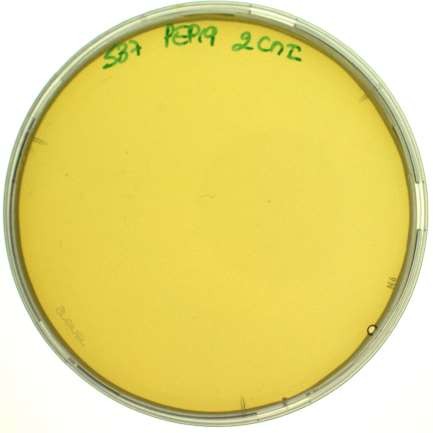

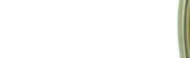

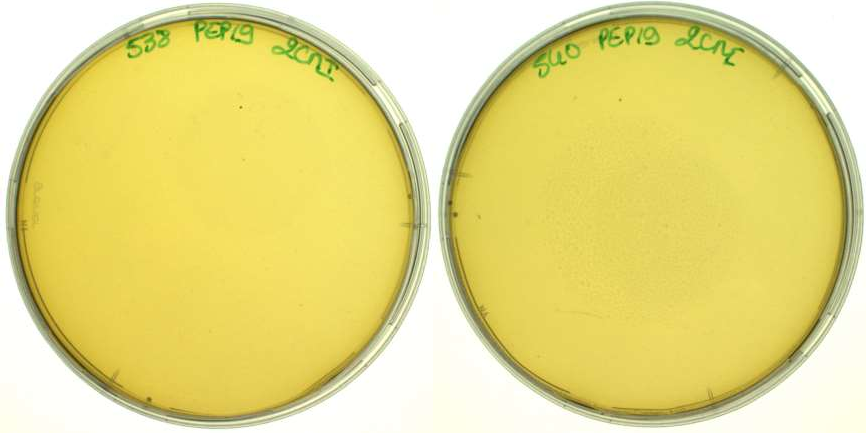

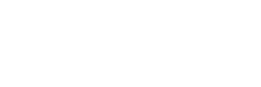

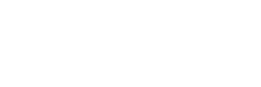

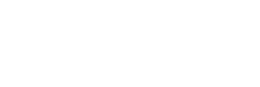


mice 1

mice 2

mice 3

BHI

1x MIC

2x MIC

Supplement: S5 Fig — Crude kidney extracts (a 105 dilution of the crude extract is used) of 3 mice were plated after 4 d of repeated treatments with Pep19. Mice are euthanatized at Day 7 and their kidneys extracted. MRSA, methicillin-resistant S. aureus. (DOCX) [file pbio.3000337.s005.docx]

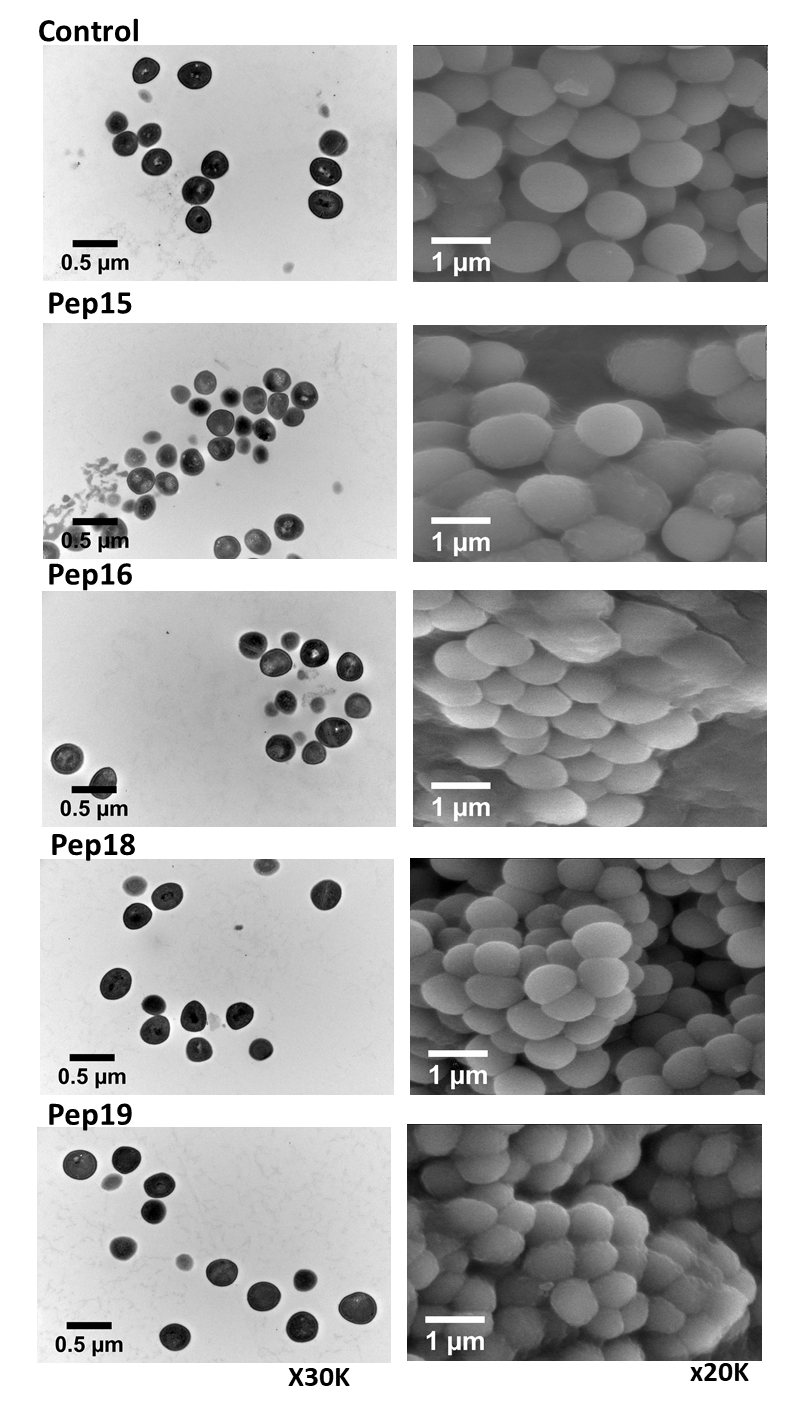

Supplement: S6 Fig — TEM (left) and SEM (right) wide field-of-view micrographs of S. aureus Newman. Shown are untreated bacteria (Control) and bacteria after treatment with Pep15, Pep16, Pep18, or Pep19 at their MICs for 2 h at 37°C. MIC, minimal inhibitory concentration; MRSA, methicillin-resistant S. aureus; SEM, scanning electron microscopy; TEM, transmission electron microscopy. (DOCX) [file pbio.3000337.s006.docx]
