# Supplementary material for: Novel antibiotics effective against gram-positive and -negative multi-resistant bacteria with limited resistance
Source: PLoS Biol. 2019 Jul 9;17(7):e3000337. doi: 10.1371/journal.pbio.3000337 (PMC6615598; doi:10.1371/journal.pbio.3000337)
Supplement: S1 Table — Clinical isolates BCB/POE and 740404 DUN are from human sepsis. The other strains are from catheter infections or suppurating wounds. Brilacidin was used as a control. MDR, multidrug resistant. (DOCX) [file pbio.3000337.s007.docx]

| **MDR *S. aureus* clinical isolates** | mg/L^-1^ | Pep15 | Pep16 | Pep18 | Pep19 | brilacidin |
| --- | --- | --- | --- | --- | --- | --- |
| PM64 | MIC | 8 | 4 | 32 | 8 | 1 |
| BCB/POE | MIC | 8 | 4 | 32 | 8 | 1 |
| COB | MIC | 8 | 2 | 32 | 2 | 1 |
| FORT | MIC | 16 | 8 | >32 | 8 | 1 |
| ROUS | MIC | 4 | 2 | 8 | 2 | 1 |
| 702697 HOU | MIC | 4 | 4 | 16 | 4 | 1 |
| 740404 DUN | MIC | 8 | 8 | 32 | 8 | 1 |
| 66534 E | MIC | 4 | 4 | 32 | 2 | 1 |
| 33845 | MIC | 16 | 8 | <32 | 16 | 1 |
