# Supplementary material for: Novel antibiotics effective against gram-positive and -negative multi-resistant bacteria with limited resistance
Source: PLoS Biol. 2019 Jul 9;17(7):e3000337. doi: 10.1371/journal.pbio.3000337 (PMC6615598; doi:10.1371/journal.pbio.3000337)
Supplement: S2 Table — The blood parameters were analyzed on Day 2 after IV administration of the peptides and pseudopeptides at 2 mg.kg−1. The mean was inferred from the measured values for 3 mice (males) per peptide/pseudopeptide. Normal values are 145–160 mmol/L for Na+, 4–7.5 mmol/L for K+, 110–120 mmol/L for Cl−, 1.2–2.8 mmol/L for inorganic phosphorus, 9.5–11 mmol/L for glucose, 9–12 mmol/L for urea, and 6–14 μmol/L for creatinine (http://www.jax.org/phenome). (DOCX) [file pbio.3000337.s008.docx]

|  | **Na^+^**  mmol/L | **K^+^**  mmol/L | **Cl^-^**  mmol/L | **I. Phos.**  mmol/L | **Glucose**  mmol/L | **Urea**  mmol/L | **Creatinine**  µmol/L |
| --- | --- | --- | --- | --- | --- | --- | --- |
| **Pep15**  Mean  SD | 153  1.1 | 3.9  0.05 | 115  1.4 | 2.3  0.4 | 11  2.4 | 7.2  1.1 | 13.7  0.9 |
| **Pep16**  Mean  SD | 155  0.9 | 3.9  0.4 | 116  1.0 | 2.8  0.25 | 10.2  1.4 | 6.6  0.6 | 14.1  1.1 |
| **Pep18**  Mean  SD | 153  1.6 | 4.6  1.3 | 114  1.1 | 2.3  0.18 | 11  0.3 | 7.9  0.8 | 13.3  0.2 |
| **Pep19**  Mean  SD | 153  0.6 | 4.4  0.64 | 115  0.6 | 2.1  0.4 | 11  0.8 | 8.2  0.2 | 10.0  0.6 |
