# Supplementary material for: Novel antibiotics effective against gram-positive and -negative multi-resistant bacteria with limited resistance
Source: PLoS Biol. 2019 Jul 9;17(7):e3000337. doi: 10.1371/journal.pbio.3000337 (PMC6615598; doi:10.1371/journal.pbio.3000337)
Supplement: S4 Table — Three endpoints were analyzed to determine the embryo development effects of peptides injected in concentrations ranging from 1 to 100 mM. Embryo body sizes and curvatures were calculated, and no toxic effects were detected. Cardiac frequency defects were observed after injection of 20 and 40 mM Pep16, and to a lesser extent with 20 mM of Pep18 and Pep19. N, number of embryos alive after treatment; p15, pseudopeptide Pep15; p16, Pep16; p18, Pep18; p19, Pep19; SD, standard deviation; SEM, standard error of the mean. (DOCX) [file pbio.3000337.s010.docx]

| **Quantitative parameters** | | | | | | |
| --- | --- | --- | --- | --- | --- | --- |
|  |  | **Mean** | **SD** | **SEM** | **N** | **95% CI** |
| **Size (µM)** | | | | | | |
|  | **Water** | 2140 | 73 | 18 | 16 | 2100 to 2178 |
|  | **nisin-1mM** | 2154 | 29 | 9 | 10 | 2133 to 2174 |
|  | **nisin-10mM** | 2102 | 133 | 42 | 10 | 2006 to 2197 |
|  | **nisin-20mM** | 2099 | 133 | 42 | 10 | 2003 to 2193 |
|  | **nisin-40mM** | 2201 | 49 | 12 | 10 | 2174 to 2226 |
|  | **p15-1mM** | 2158 | 41 | 13 | 10 | 2128 to 2187 |
|  | **p15-10mM** | 2154 | 51 | 16 | 10 | 2116 to 2190 |
|  | **p15-20mM** | - | - | - | - | - |
|  | **p15-40mM** | - | - | - | - | - |
|  | **p16-1mM** | 2176 | 39 | 12 | 10 | 2148 to 2203 |
|  | **p16-10mM** | 2151 | 51 | 16 | 10 | 2114 to 2188 |
|  | **p16-20mM** | 2116 | 56 | 28 | 4 | 2027 to 2205 |
|  | **p16-40mM** | 2223 | 47 | 33 | 2 | 1797 to 2648 |
|  | **p18-1mM** | 2139 | 39 | 12 | 10 | 2110 to 2166 |
|  | **p18-10mM** | 2154 | 31 | 10 | 10 | 2131 to 2176 |
|  | **p18-20mM** | 2155 | 33 | 12 | 8 | 2127 to 2183 |
|  | **p18-40mM** | - | - | - | - | - |
|  | **p19-1mM** | 2157 | 56 | 18 | 10 | 2117 to 2197 |
|  | **p19-10mM** | 2127 | 45 | 14 | 10 | 2094 to 2159 |
|  | **p19-20mM** | 2120 | 51 | 26 | 4 | 2038 to 2201 |
|  | **p19-40mM** | - | - | - | - | - |
| **Curvature (°)** | | | | | | |
|  | **Water** | 179 | 1 | 0 | 16 | 178 to 179 |
|  | **nisin-1mM** | 179 | 1 | 0 | 10 | 178 to 179 |
|  | **nisin-10mM** | 179 | 1 | 0 | 10 | 178 to 179 |
|  | **nisin-20mM** | 179 | 1 | 0 | 10 | 178 to 179 |
|  | **nisin-40mM** | 179 | 0 | 0 | 10 | 178 to 179 |
|  | **p15-1mM** | 179 | 1 | 0 | 10 | 178 to 179 |
|  | **p15-10mM** | 179 | 1 | 0 | 10 | 178 to 179 |
|  | **p15-20mM** | - | - | - | - | - |
|  | **p15-40mM** | - | - | - | - | - |
|  | **p16-1mM** | 180 | 1 | 0 | 10 | 179 to 180 |
|  | **p16-10mM** | 180 | 1 | 0 | 10 | 179 to 180 |
|  | **p16-20mM** | 180 | 1 | 0 | 4 | 178 to 180 |
|  | **p16-40mM** | 179 | 1 | 1 | 2 | 172 to 184 |
|  | **p18-1mM** | 179 | 0 | 0 | 10 | 178 to 179 |
|  | **p18-10mM** | 179 | 1 | 0 | 10 | 178 to 179 |
|  | **p18-20mM** | 179 | 1 | 0 | 8 | 178 to 179 |
|  | **p18-40mM** | - | - | - | - | - |
|  | **p19-1mM** | 179 | 0 | 0 | 10 | 178 to 179 |
|  | **p19-10mM** | 179 | 1 | 0 | 10 | 178 to 179 |
|  | **p19-20mM** | 179 | 1 | 0 | 4 | 177 to 180 |
|  | **p19-40mM** | - | - | - | - | - |
| **Cardiac frequency (heart beat/10 sec)** | | | | | | |
|  | **Water** | 30 | 0 | 0 | 16 | 30 to 31 |
|  | **nisin-1mM** | 29 | 1 | 0 | 10 | 28 to 30 |
|  | **nisin-10mM** | 30 | 1 | 0 | 10 | 29 to 30 |
|  | **nisin-20mM** | 30 | 1 | 0 | 10 | 29 to 30 |
|  | **nisin-40mM** | 30 | 0 | 0 | 10 | 29 to 30 |
|  | **p15-1mM** | 30 | 0 | 0 | 10 | 29 to 30 |
|  | **p15-10mM** | 30 | 0 | 0 | 10 | 29 to 30 |
|  | **p15-20mM** | - | - | - | - | - |
|  | **p15-40mM** | - | - | - | - | - |
|  | **p16-1mM** | 30 | 0 | 0 | 10 | 30 to 31 |
|  | **p16-10mM** | 31 | 1 | 0 | 10 | 30 to 31 |
|  | **p16-20mM** | 6 | 8 | 4 | 4 | 0 to 18 |
|  | **p16-40mM** | 0 | 0 | 0 | 2 | 0 |
|  | **p18-1mM** | 29 | 1 | 0 | 10 | 28 to 30 |
|  | **p18-10mM** | 29 | 1 | 0 | 10 | 28 to 30 |
|  | **p18-20mM** | 22 | 13 | 5 | 8 | 10 to 33 |
|  | **p18-40mM** | - | - | - | - | - |
|  | **p19-1mM** | 30 | 1 | 0 | 10 | 29 to 30 |
|  | **p19-10mM** | 30 | 1 | 0 | 10 | 29 to 30 |
|  | **p19-20mM** | 21 | 10 | 5 | 4 | 4 to 37 |
|  | **p19-40mM** | - | - | - | - | - |
