# Supplementary material for: Novel antibiotics effective against gram-positive and -negative multi-resistant bacteria with limited resistance
Source: PLoS Biol. 2019 Jul 9;17(7):e3000337. doi: 10.1371/journal.pbio.3000337 (PMC6615598; doi:10.1371/journal.pbio.3000337)
Supplement: S5 Table — Clinical signs observed on Day 1 after IV administration of each of the peptide and pseudopeptides in sets of 3 mice. Not observed symptoms are marked with a dash. (DOCX) [file pbio.3000337.s011.docx]

|  | Prostration | - | - | - | - |  | 1 |
| --- | --- | --- | --- | --- | --- | --- | --- |
|  | Red-colored ears | - | - | - | 3 |  | 1 |
|  | Red-colored eyes | - | - | - | 3 |  | - |
|  | Staggering gait | - | - | - | - |  | 2 |
|  | Soiled urogenital region | - | - | - | - |  | - |
|  | Sudden startle | 1 | - | - | 3 |  | 2 |
|  | Ventral recumbency | - | - | - | - |  | - |
|  | Hindlimbs widespread | - | - | - | 2 |  | 1 |
|  | Injected Pep19 (mg/Kg) | 1.5 | 2 | 2.5 | 5 (bolus) |  | 5 (slow injection) |
|  | Abdominal breathing | 2 | - | - | - |  | - |
|  | Decreased grasping reflex | - | - | - | 2 |  | - |
|  | Dyspnea | - | - | 3 | 3 |  | - |
|  | Half-closed eyes | - | - | - | - |  | - |
|  | Hunched posture | - | 1 | 1 | 3 |  | - |
|  | Hypoactivity | - | 1 | 3 | 3 |  | 2 |
|  | Hypotonia | - | - | - | 2 |  | - |
|  | Piloerection | 3 | 3 | 3 | 3 |  | 3 |
|  | Red-colored ears | - | - | - | 3 |  | 2 |
|  | Red-colored eyes | - | - | - | 3 |  | - |
|  | Staggering gait | - | 1 | - | - |  | 3 |
|  | Sudden startle | 1 | - | - | 3 |  | - |
|  | Hindlimbs widespread | - | - | - | 1 |  | 1 |
|  |  |  |  |  |  |  |  |
