# Supplementary material for: Novel antibiotics effective against gram-positive and -negative multi-resistant bacteria with limited resistance
Source: PLoS Biol. 2019 Jul 9;17(7):e3000337. doi: 10.1371/journal.pbio.3000337 (PMC6615598; doi:10.1371/journal.pbio.3000337)
Supplement: S7 Table — Degradation kinetics of the 4 cyclic peptides in human or mouse sera (peptide concentration: 10−4 M) and compared to 3 peptide reference antibiotics (nisin, polymyxin B, and daptomycin). Peptide amounts and half-lives were determined by HPLC. Experiments were conducted on 25% human or mouse sera. Values are shown in minutes. The mean values correspond to biological triplicates. HPLC, high pressure liquid chromatography. (DOCX) [file pbio.3000337.s013.docx]

|  | nisin | polymyxin B | daptomycin | Pep15 | Pep16 | Pep18 | Pep19 |
| --- | --- | --- | --- | --- | --- | --- | --- |
| **Human** | 22 ±5 | 1380 ±200 | 3136 ±400 | 2320  ± 240 | 1442  ±170 | 980  ±100 | 1450  ±260 |
| **Mouse** | 7 ±2 | 640 ±162 | > 3500 | 1080  ±90 | 990  ±150 | 510  ±150 | 540  ±105 |
