# Supplementary material for: Novel antibiotics effective against gram-positive and -negative multi-resistant bacteria with limited resistance
Source: PLoS Biol. 2019 Jul 9;17(7):e3000337. doi: 10.1371/journal.pbio.3000337 (PMC6615598; doi:10.1371/journal.pbio.3000337)
Supplement: S8 Table — (DOCX) [file pbio.3000337.s014.docx]

| **AA** | **HN** | **HA** | **HB** | **Others** |
| --- | --- | --- | --- | --- |
| **F1** | 7.35 | 4.47 | 2.88 | HD 7.07; HE 7.27; HZ 7.13 |
| **F2** | 7.62 | 4.46 | 2.88; 3.00 | HD 7.11; HE 7.19; HZ 7.26 |
| **W3** | 7.80 | 4.74 | 3.16; 3.40 | HD1 7.42; HE1 9.95; HE3 7.71; HH2 7.18 HZ2 7.51; HZ3 7.13 |
| **R4** | 8.14 | 4.14 | 1.73; 1.87 | HG 1.21; 1.35; HD 3.01; HE 6.94 |
| **R5** | 7.83 | 4.27 | 1.85, 1.96 | HG 1.67; HD 3.21; HE 7.16 |
| **V6** | 7.86 | 4.18 | 2.27 | HG 0.97 |
| **K7** | 7.93 | 3.97 | 1.51, 1.64 | HG 1.06;1.13; HD 1.58 |
