# Supplementary material for: Novel antibiotics effective against gram-positive and -negative multi-resistant bacteria with limited resistance
Source: PLoS Biol. 2019 Jul 9;17(7):e3000337. doi: 10.1371/journal.pbio.3000337 (PMC6615598; doi:10.1371/journal.pbio.3000337)
Supplement: S9 Table — (DOCX) [file pbio.3000337.s015.docx]

| **AA** | **HN** | **HA** | **HB** | **Others** |
| --- | --- | --- | --- | --- |
| **Nal1** | 9.32 |  | 4.17; 4.44 | HD1 7.39; HE1 7.70; HZ1 7.38; HG2 8.13; HD3 7.39; HE3 7.32 HZ2 7.72; HS 3.80; 3.89 |
| **F2** | 7.96 | 3.74 | 1.93; 2.38 | HD 6.87; HE 6.95; HZ 6.79 |
| **Nal3** | 9.00 |  | 3.64; 3.97 | HD1 7.34; HE1 7.65; HZ1 7.33; HG2 8.03; HD3 7.45; HE3 7.45 HZ2 7.73; HS 3.39; 3.50. |
| **R4** | 8.19 | 3.88 | 0.41; 1.27 | HG 0.28; 0.41; HD 2.51; HE 6.58 |
| **R5** | 7.75 | 4.15 | 1.75, 1.83 | HG 1.64; HD 3.12 ; HE 7.14 |
| **V6** | 7.86 | 3.57 | 1.90 | HG 0.81 |
| **K7** | 7.33 | 4.04 | 1.44 | HG 1.09; HD 1.49; HE 2.81 |
