# Supplementary material for: Novel antibiotics effective against gram-positive and -negative multi-resistant bacteria with limited resistance
Source: PLoS Biol. 2019 Jul 9;17(7):e3000337. doi: 10.1371/journal.pbio.3000337 (PMC6615598; doi:10.1371/journal.pbio.3000337)
Supplement: S10 Table — SDS, sodium dodecyl sulfate. (DOCX) [file pbio.3000337.s016.docx]

| Conformational restraints  Total inter residues  Restraint violations  Distance restraints > 0.3 Å  Energetics statistics (kcal.mol^-1^)  E_tot_  Average RMSD (pairwise, Å)  Backbone  All heavy atoms | Pep18  50  0  -391 ± 2  0.11  1.09 | Pep19  109  0  -144 ± 1  0.10  0.82 |
| --- | --- | --- |
