# Supplementary material for: Novel antibiotics effective against gram-positive and -negative multi-resistant bacteria with limited resistance
Source: PLoS Biol. 2019 Jul 9;17(7):e3000337. doi: 10.1371/journal.pbio.3000337 (PMC6615598; doi:10.1371/journal.pbio.3000337)
Supplement: S11 Table — SDS, sodium dodecyl sulfate. (DOCX) [file pbio.3000337.s017.docx]

| **Residues** | **Torsion angles (°)** | | | | | | |
| --- | --- | --- | --- | --- | --- | --- | --- |
|  | **φ** | **θ** | | **ψ** | |  |  |
| Nal 1 | 109.0 ± 5 | | -133.3 ± 3 | | 59.9 ± 2 | |  |
| Phe 2 | -141.3 ± 4 | | - | | 129.5 ± 5 | |  |
| Nal 3 | 101.2 ± 5 | | -96.2 ± 10 | | -3.5 ± 16 | |  |
| Arg 4 | -149.5 ± 4 | | - | | -6.5 ± 4 | |  |
| Arg 5 | -68.0 ± 2 | | - | | -23.1 ± 5 | |  |
| Val 6 | -80.0 ± 2 | | - | | 55.7 ± 3 | |  |
| Lys 7 | -145.1 ± 4 | | - | | 160.6 ± 9 | |  |
|  |  | |  | |  | |  |
