# Supplementary material for: Novel antibiotics effective against gram-positive and -negative multi-resistant bacteria with limited resistance
Source: PLoS Biol. 2019 Jul 9;17(7):e3000337. doi: 10.1371/journal.pbio.3000337 (PMC6615598; doi:10.1371/journal.pbio.3000337)
Supplement: Supplementary file 14 [file pbio.3000337.s009.docx]

Qualitative parameters																				
	Mortality		Cardiac		Blood flow		Cerebelar		Necrosis		Pericardial		Otic vesicle		Motility		Brain defect			
	F	P	F	P	F	P	F	P	F	P	F	P	F	P	F	P	F	P	N	CP
**water**	0	0	0	0	0	0	0	0	0	0	0	0	0	0	0	0	0	0	16	0
**nisin-1mM**	0	0	0	0	0	0	0	0	0	0	0	0	0	0	0	0	0	0	10	0
**nisin-10mM**	0	0	0	0	0	0	0	0	0	0	0	0	0	0	0	0	0	0	10	0
**nisin-20mM**	0	0	0	0	0	0	0	0	0	0	0	0	0	0	0	0	0	0	10	0
**nisin-40mM**	0	0	0	0	0	0	0	0	0	0	0	0	0	0	0	0	0	0	10	0
**p15-1mM**	0	0	0	0	0	0	0	0	0	0	0	0	0	0	0	0	0	0	10	0
**p15-10mM**	0	0	0	0	0	0	0	0	0	0	0	0	0	0	0	0	0	0	10	0
**p15-20mM**	1	100	-	-	-	-	-	-	-	-	-	-	-	-	-	-	-	-	10	100
**p15-40mM**	1	100	-	-	-	-	-	-	-	-	-	-	-	-	-	-	-	-	10	100
**p16-1mM**	0	0	0	0	0	0	0	0	0	0	0	0	0	0	0	0	0	0	10	0
**p16-10mM**	0	0	0	0	0	0	0	0	0	0	0	0	0	0	0	0	0	0	10	0
**p16-20mM**	0.6	60	0	0	0.4	40	0	0	0	0	0.4	40	0	0	0.2	20	0	0	10	160
**p16-40mM**	0.8	80	0	0	0.1	10	0	0	0	0	0	0	0.1	10	0	0	0.1	10	10	110
**p18-1mM**	0	0	0	0	0	0	0	0	0	0	0	0	0	0	0	0	0	0	10	0
**p18-10mM**	0	0	0	0	0	0	0	0	0	0	0	0	0	0	0	0	0	0	10	0
**p18-20mM**	0.2	20	0	0	0.2	20	0	0	0.2	20	0.2	20	0	0	0.2	20	0	0	10	100
**p18-40mM**	1	100	-	-	-	-	-	-	-	-	-	-	-	-	-	-	-	-	10	100
**p19-1mM**	0	0	0	0	0	0	0	0	0	0	0	0	0	0	0	0	0	0	10	0
**p19-10mM**	0	0	0	0	0	0	0	0	0	0	0	0	0	0	0	0	0	0	10	0
**p19-20mM**	0.6	60	0	0	0.2	20	0	0	0	0	0.2	20	0	0	0.3	30	0	0	10	130
**p19-40mM**	1	100	-	-	-	-	-	-	-	-	-	-	-	-	-	-	-	-	10	100
