# Supplementary material for: Novel antibiotics effective against gram-positive and -negative multi-resistant bacteria with limited resistance
Source: PLoS Biol. 2019 Jul 9;17(7):e3000337. doi: 10.1371/journal.pbio.3000337 (PMC6615598; doi:10.1371/journal.pbio.3000337)
Supplement: Supplementary file 15 [file pbio.3000337.s012.docx]

**Pep15** injected doses (mg/kg)	1.5	2	2.5	5 (bolus)	5 (slow injection)
White blood cells (g/L)	1.6 (±0.4)	1.9 (±0.3)	3.8 (±1.3)	9.2 (±0.5)	5.9 (±3.8)
Neutrophils (g/L)	0.3 (±.04)	0.35 (±.03)	0.6 (±0.2)	7.2 (±1.7)	4.6 (±3.5)

**Pep16** injected doses (mg/kg)	1.5	2	2.5	5 (bolus)	5 (slow injection)
White blood cells (g/L)	1.3 (±0.1)	1.5 (±0.4)	2.3 (±0.4)	2.6 (±0.2)	1.8 (±0.5)
Neutrophils (g/L)	0.3 (±0.1)	0.2 (±0.06)	0.6 (±0.15)	1.3 (±0.7)	0.6 (±0.2)

**Pep18** injected doses (mg/kg)	1.5	2	2.5	5 (bolus)	5 (slow injection)
White blood cells (g/L)	1.8 (±0.7)	1.3 (±0.1)	2.3 (±0.6)	4.6 (±5.6)	5.7 (±5.4)
Neutrophils (g/L)	0.4 (±0.2)	0.25 (±0.1)	0.49 (±0.2)	3.7 (±5.1)	4.32 (±4.8)

**Pep19** injected doses (mg/kg)	1.5	2	2.5	5 (bolus)	5 (slow injection)
White blood cells (g/L)	1.8 (±0.2)	1.6 (±0.9)	2.3 (±0.4)	2.4 (±0.03)	2.8 (±0.7)
Neutrophils (g/L)	0.4 (±0.1)	0.4 (±0.2)	0.4 (±0.1)	0.7 (±0.16)	1.2 (±0.8)
